# An Integrated Strategy for Sustainable Dioxin Remediation: Sources, Places of Contamination, and Toxicity

**DOI:** 10.3390/molecules31101705

**Published:** 2026-05-18

**Authors:** Muhammad Hubab, Afrah Siddique, Sami Sayadi, Mohammed Abu-Dieyeh, Roda Al-Thani, Lama Soubra, Mohammad A. Al-Ghouti

**Affiliations:** 1 Department of Biological and Environmental Sciences, College of Arts and Sciences, Qatar University, Doha P.O. Box 2713, Qatar; mh2300810@student.qu.edu.qa (M.H.); afrah@qu.edu.qa (A.S.); dandelion@qu.edu.qa (M.A.-D.); ralthani@qu.edu.qa (R.A.-T.); lama.soubra@qu.edu.qa (L.S.); 2 Center for Sustainable Development, College of Arts and Sciences, Qatar University, Doha P.O. Box 2713, Qatar; ssayadi@qu.edu.qa

**Keywords:** dioxin, soil contamination, incineration, human health, environmental pollution, biodegradation, aryl hydrocarbon receptor

## Abstract

Dioxins are highly persistent organic pollutants that exist in soil. Their hydrophobic and lipophilic characteristics facilitate long-term stability, posing high risks to the ecosystem and human health. They can be released by different sources, such as the incineration of waste materials, industrial activities, the production of pesticides, and natural or accidental events like forest fires. Dioxins accumulate in food chains and persist in the environment because dioxins are less volatile as well as chemically stable and can strongly bind to organic matter. The accumulation and persistence of dioxins in aquatic and terrestrial systems make them a significant threat to the environment, even at very low concentrations. This review explains the key sources of dioxin-contaminated soil, including industrial emissions and atmospheric deposition, and assesses the associated risks. The transport, places of contamination, and overall status of dioxins are also highlighted in this study. The review also examines the mechanisms of dioxin toxicity, focusing on their interference with hormonal functions and gene expression, as mediated through the aryl hydrocarbon receptor (AhR). This AhR activation leads to gene responses and causes immunotoxicity, endocrine disruption, and oxidative stress. Furthermore, various remediation strategies like biological, physical, and chemical remediation are discussed here as effective approaches for reducing ecological and health risks and promoting soil sustainability.

## 1. Introduction

**Figure 1:**
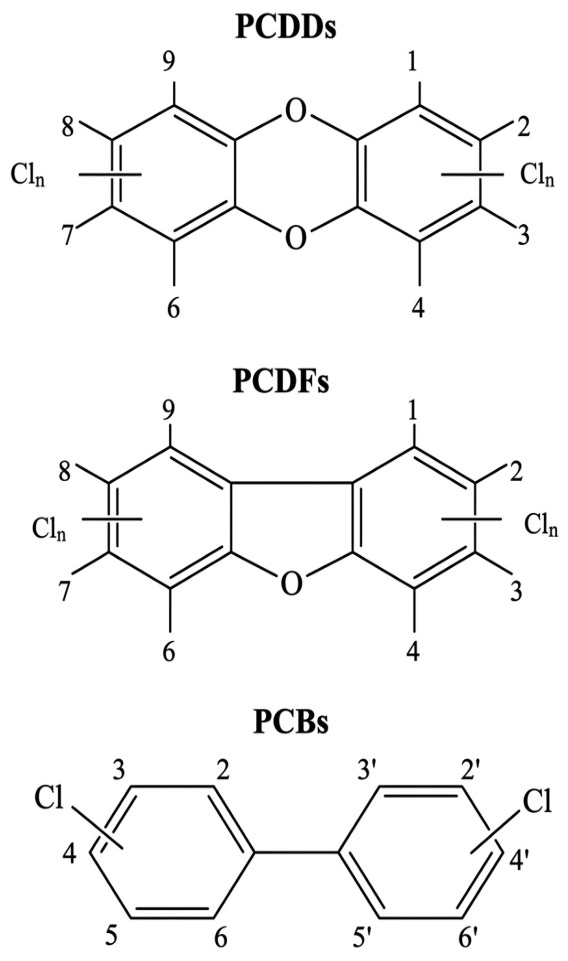
The structures of polychlorinated dibenzo-p-dioxins (PCDDs), polychlorinated dibenzofurans (PCDFs), and PCBs [4].

Dioxins are highly stable compounds with a solid nature, high boiling and melting points, and low vapor pressure. They have very low solubility in water and possess excellent thermal stability, as they are degraded completely above 1200 °C. Dioxins are also resistant to both strong alkalis and acids and tend to accumulate on organic materials, mainly in soil [1]. Dioxins are undesirable pollutants that are primarily produced unintentionally during human activities; the sources include industrial pollution, incineration, pulp and paper production, and the production of fungicides, herbicides, and pesticides. Dioxins are highly stable compounds and can be characterized by their nonpolar, hydrophobic, and lipophilic nature. Dioxins are categorized into three main groups: coplanar polychlorinated biphenyls (dioxin-like PCBs or dl-PCBs), polychlorinated dibenzo-p-dioxins (PCDDs, commonly known as dioxins), and polychlorinated dibenzofurans (PCDFs, known as furans) [2]. Figure 1 shows the chemical structures of PCBs, PCDFs, and PCDDs. The pollutants are known for their long-term persistence in the ecosystem, toxicity, and hazardous effects on both the ecosystem and humans. 2,3,7,8-tetrachlorodibenzo-p-dioxin (TCD) is considered very toxic and a prevalent form of dioxin [3].

Though dioxins can be produced by natural activities such as fires in forests and eruptions, different human actions and activities have also been considered the main source of such chemicals over the last two centuries. In the past, industrialization was considered a major contributor, but many industrial processes are now regulated. As an example, the uncontrolled burning of waste from domestic sources in open fires is a more important concern. Dioxins are of specific concern due to their poisonous effects and the wide range of health problems they can cause. Dioxin levels are widely detected in the human population, especially in people who live in industrialized countries [5]. Different studies have reported dioxins as the main environmental pollutants in soil; various sources, associated risks, and mitigation strategies are given in Table 1. These pollutants persist in the soil for a long time, making this class of environmental pollutants one of the most severe. Human populations are exposed to dioxins mostly through the ingestion of contaminated food, which is considered the main route of entry. Contamination arises from various sources, like past military activities, industrialization, and chemical production [6].

Exposure to dioxins in the human population has been related to different harmful health effects, including weak immunity, neurodevelopmental issues, and thyroid and steroid disruptions. Developmental impacts are considered to be the most sensitive health concern in children, with breastfeeding neonates being the most vulnerable. These substances can travel from their emission source and can accumulate in food chains. The concentration of dioxins increases at higher levels of the food chain through biomagnification. Consumption of contaminated food is a primary route of human exposure to dioxins. Public regulatory and health measures are essential to minimize the emissions of these substances, as mandated by the Stockholm Convention, as well as to decrease human exposure, especially in children [15].

The 2020 COVID-19 pandemic intensified the excessive use of personal protective equipment, the improper disposal of which leads to the accumulation of dioxins in the environment [16]. This increased research interest in pandemic-driven pollution. Dioxins are now recognized as persistent, bio-accumulative toxicants that penetrate trophic levels. Dietary exposure is the main cause of dioxin intake in humans. Recent studies highlight the adverse health outcomes of dioxins, including endocrine disruption, metabolic disorders, cardiovascular diseases, neurotoxicity, and immune dysfunction, in addition to their carcinogenic effects. Recent epidemiological and toxicological studies report associations between PCDDs and increased risks of diabetes, coronary heart disease, and reproductive impairments, while also emphasizing their long biological half-life and accumulation in adipose tissues [17]. Mechanistic toxicology-based studies demonstrate complex molecular pathways, including AhR-mediated responses, which contribute to multi-system toxicity, even at low exposure levels [18].

Dioxins are persistent environmental pollutants, often referred to as “repeat offenders” for repeatedly causing harm. They are part of the “dirty dozen,” a group of lethal chemicals recognized for their persistence in the ecosystem. The International Agency for Research on Cancer designated them the most carcinogenic compound. Once the dioxins enter the body or the environment, they remain because of their ability to dissolve fats and their strong chemical stability. Normally, their half-life in the human body is seven years [19]. As dioxins are highly persistent in the environment for such long periods, they penetrate the soil and sediments. The dioxin compound transports into terrestrial and marine environments, where they biomagnify in the food chain, resulting in high concentrations in humans. This causes a serious risk for future generations [20]. Dioxin compounds were recognized for the first time in the late 19th century, but common industrial production began in the 20th century. In 1929, PCBs started being produced in Alabama, and dioxin substances were produced as by-products. Dioxins gained significant attention due to their health impacts, particularly with the use of Agent Orange in the Vietnam War (1960–1972), which led to widespread contamination [21].

Additionally, environmental disasters like that at Seveso, Italy, a chemical plant explosion in 1976, and the contamination of Times Beach, Missouri, drew public awareness to the dangers of dioxins [21]. Even trace amounts, such as one part per million, can cause major infections [22]. Dioxin consists of 75 PCDD and 135 PCDF congers, variable in toxicity based on the arrangement and number of chlorine atoms. Out of 210 total congers, 17 are mainly harmful because of the chlorination at specific positions, namely, 2, 3, 7, and 8 on the aromatic ring. Among the 2,3,7,8-substituted, TCDD and TCDF are recognized as the most toxic, according to the (I-TEF) international toxicity equivalence factor system [23]. Various studies have been conducted on different remediation techniques for dioxin, such as physical, chemical, and biological means. The purpose of biological remediation is the removal of persistent organic compounds from dioxin-contaminated soil by using different microorganisms and enzymes. The procedure is comparatively beneficial and eco-friendly. Dioxin can be removed through microbial activities, in both aerobic and anaerobic conditions. Various microorganisms utilize dioxins as a source of energy and carbon. Furthermore, the processes of phytoremediation have shown significant potential, mainly when a plant with considerable biomass is used. Dioxin can be absorbed efficiently by plants from the soil, and this has been shown in the laboratory [24].

Dioxins have been extensively studied for their toxicity and persistence. Most of the studies focus primarily on their remediation procedures and environmental behavior. This study aims to provide a comprehensive review of various dioxin sources, the food chain transfer, soil contamination and transport, and the mechanisms of molecular toxicity, as well as techniques for remediation in the soil. Dioxin is identified as a major pollutant in soil by linking the contamination to AhR-related health risks and evaluating the different integrated mitigation techniques [25]. Although the persistence and toxicity of dioxins are thoroughly understood, successful management cannot be achieved without knowledge of their chemical nature. Sustainable soil restoration requires a holistic approach that connects the sources, the environmental behavior, and the health effects to a specific remedial intervention. The novelty of this review lies in the integration of dioxin sources and food chain transfer review and also offers toxicity related to AhR. It also provides a step-based framework to be used for selecting and implementing a suitable remediation strategy based on site-specific characteristics.

**Figure 2:**
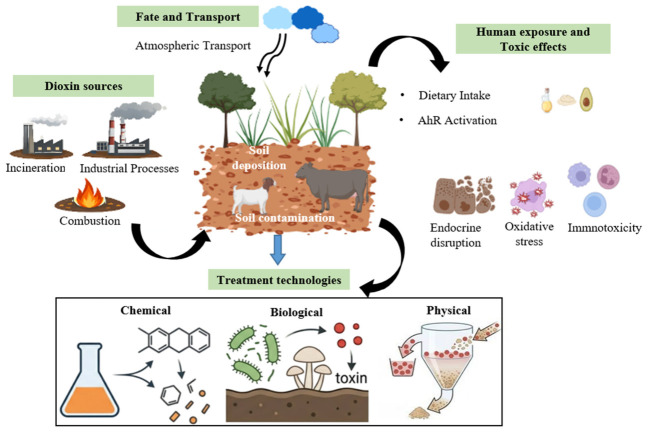
Integrated model of dioxin sources, environmental sites of contamination and transport, different pathways of exposure, toxic effects, and different remediation technologies. BioRender was used for figure creation. BioRender: Scientific Figure and Illustration Software.

This review shows a cohesive conceptual framework that integrates the sources of dioxins, places of contamination, pathways of exposure, and mechanisms of toxicity to select the effective strategies for remediation, as shown in Figure 2. Dioxins are released primarily from different human activities such as incineration, combustion, and industrial processes. After they are released into the environment, dioxins undergo long-range atmospheric transport and are deposited into the soil [5].

After the deposition in the soil, dioxin enters the food chain through plant uptake and subsequently accumulates in the tissues of animals, leading to human exposure, commonly through intake of dietary products. Dioxin causes toxicity through the activation of AhR, in which alteration in gene expression occurs, and this causes disruption of endocrine levels, oxidative stress, and immunotoxicity. These connected procedures collectively determine the distribution of environmental pollutants and the risks associated with human health [26]. Remediation and restoration techniques should be applied for the quality improvement of dioxin-contaminated soil. Following the excavation of dioxin-contaminated soil, the biological, chemical and physical remediation technologies are used for soil treatment. These techniques may be applied in situ or ex situ. Usually, the cleanup procedures involve the removal of contaminants from the affected area or making them less harmful [27].

## 2. Sources, Transport, and Locations of Dioxins in Soil

**Figure 3:**
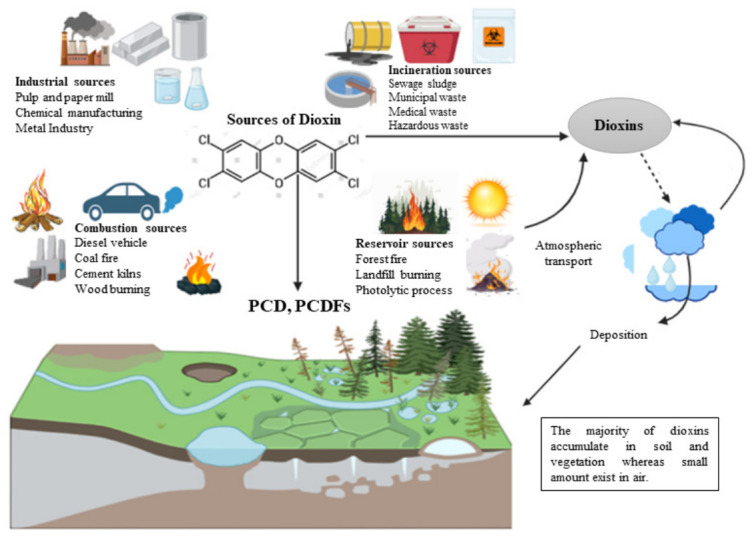
Various natural and anthropogenic sources of dioxin release and the grasshopper effect on the environment. BioRender was used for figure creation. BioRender: Scientific Figure and Illustration Software.

Dioxins usually exist in both human-made and natural environments. These compounds are a major example of persistent organic pollutants (POPs), known for their highly complex structures as well as varying degrees of toxicity. Historical analysis of human tissue samples reveals significantly lower levels of dioxins in the past, in comparison to modern times. Currently, the dioxin-like compounds present in the ecosystem are primarily a result of human activities. These substances are presented through several pathways and in differing amounts, depending on the source. Their widespread distribution indicates the presence of many sources and the possibility of long-range transport. The main sources of dioxin release into the environment have been categorized into four primary groups. The dioxins present in the soil because of human activities pose long-term health and ecological risk, with bioremediation methods like phytoremediation offering mitigation, as depicted in Figure 3 [28].

Different factors affect the transport and contamination of dioxin in the soil, including different soil properties like moisture, pH, organic matter, and texture, along with the environmental conditions, including surface characteristics, vegetation, groundwater, biological activities, and weather. The soil particles most often absorb them because of their low mobility and biodegradability [29]. Once dioxins are released into the air, they can travel long distances through atmospheric transport, eventually depositing in soils far from the initial sources of emission. This process, often referred to as the “grasshopper effect,” involves dioxins being volatilized into the air, carried by air currents, and then redeposited onto the soil during subsequent weather events like rainfall. This movement is affected by different environmental factors, like wind patterns, precipitation, and the hydrological cycle [30]. Dioxins are mainly released into the atmosphere, and carried over long distances, and they finally settle on the water surface and soil. The hydrophobic nature of dioxins binds them strongly to organic matter in soil and sediments. This hydrophobicity leads to the high persistence of dioxins, with environmental half-lives that can extend from decades to centuries, depending on the conditions of the soil [31].

The United Nation Environmental Programme (UNEP) recently identified the main factors influencing the contamination and transport of dioxin in the ecological system in light of different climate changes: rainfall; pollutant transporting by living organisms; secondary sources releasing pollutant back into the environment; flow of water; transport and degradation of POPs; severe weather conditions; melting of mountain glaciers; and wind speed. Different climate changes led to exposure to such pollutants through different mechanisms, sources, and processes [32].

The processes of incineration are considered the most significant and largest contributors to dioxin emissions into the environment. These harmful compounds are produced and released during various types of incineration activities. Incineration of public solid waste is considered to be the primary source of dioxin release into the environment. The mechanisms responsible for their formation have been extensively studied by various researchers. The furans and dioxins have been noted to be primarily released into the environment through two main surface catalytic processes: (i) formation through de novo synthesis and (ii) formation from precursors. Furthermore, various previous studies have reported the existence of high amounts of dioxins and their precursors in public solid waste, with concentrations averaging about 50 ng International Toxic Equivalent per kilogram (I-TEQ/kg) [33]. Different materials are included in hospital waste, such as cell and tissue culture, bandages, test tubes, human organs, blood tubes, different types of needles and syringes, and various plastic materials. This modern period is mostly known as the plastic age. In every country, the process of incineration has been considered the most commonly used treatment of hospital waste on a large scale. But these incinerators, which are numerous and typically burn waste with high chlorine contents, do not use advanced technologies, making them a significant source for the emission of dioxin [34]. Hazardous waste refers to the harmful byproducts of industrial chemical processes. Depending on the composition, this waste can exhibit properties such as explosiveness, oxidizing potential, flammability, corrosiveness, infectivity, mutagenicity, irritation, toxicity, or carcinogenicity. To address the unique risks posed by such waste, dedicated incineration practices for hazardous materials were introduced several years ago. This method is particularly effective for hazardous organic compounds, including chlorinated phenols [35]. The sewage sludge, a solid byproduct rich in toxic metals and organic matter, is generated during wastewater treatment processes. Because of the restrictions on landfill use, the planned prohibition of sea disposal, and challenges in recycling, incineration has become a widely adopted method for sewage sludge disposal. Although limited in number, some studies have explored the processes and implications of sewage sludge incineration [36].

Sources of combustion refer to processes where materials are burned, releasing different pollutants into the atmosphere, which leads to the dispersal of dioxin. The practice of using harmful waste as an alternative fuel in cement kilns has raised concerns among both organizations and individuals. Nearly 16% of cement services use toxic waste for this purpose. The limited data show that the levels of PCDD in clinker dust and stack emissions from these kilns might be considerably higher compared to kilns that do not burn toxic waste [37]. Biomass combustion, mainly the residential burning of wood in developing countries, contributes to the emission of dioxins, with high levels being associated with the use of inefficient stoves and contaminated fuel sources [38].

Wood burning has been recognized as a major dioxin source, with studies detecting these compounds in the smoke and residue from such fires in non-industrial settings. The findings of the European Emission Inventory suggest combustion of wood is the primary contributor to dioxin emissions in the air. A detailed review suggests that emissions of dioxin from the burning of wood amount to around 945 g I-TEQ/year [39]. Recent studies on industrial boilers fueled by biomass show that combustion is a major source of PCDD/F emissions. The measured concentration was between 0.049 and 12.7 ng Nm^−3^ (at 11% O_2_), while I-TEQ values reached up to 1.71 ng I-TEQ Nm^3^ [40].

According to one study, there is limited research on dioxin emissions from diesel vehicles. Studies conducted in Norway and Sweden have examined this issue. Cremation processes can generate both organic materials and chlorine, making crematoria potential sources of dioxin emissions. According to inventory estimates, crematoria contribute about 0.3% of dioxin emissions in Europe and 0.24% in the United States. While dioxin emissions from coal-fired services are relatively lower than those from wood burning, their widespread presence, large size, and tall stacks suggest they may impact extensive areas [7].

Different industrial activities lead to the production of dioxin. For example, the production of bleached pulp and paper has historically led to the release of dioxins into paper products, soil, and water. Dioxins can form during the chlorination process involving the natural phenolic compounds found in wood pulp. Research shows that a pulp mill in China generated waste containing dioxins at a concentration of 30 picograms (pg)/L I-TEQ [41]. Chemical production also plays an important role in dioxin production. Dioxin is produced as an unintended byproduct during the production of chlorinated chemicals, including phenoxy herbicides, chlorinated phenols, PCBs, chlorinated benzenes, halogenated diphenyl ethers, chlorinated aliphatic compounds, and chlorinated catalysts. While the manufacturing of several chlorinated phenolic intermediates and PCBs was halted in the United States by the late 1970s, global production continued until 1990. Moreover, the ongoing disposal of these substances and limited use can still lead to the emission of dioxins into the environment [42].

### Worldwide Patterns of Dioxin Contamination

Global emissions and deposition patterns reveal that dioxins do not necessarily accumulate in the areas where they are produced. Even regions with minimal or no active dioxin emissions can still receive considerable amounts of these compounds due to long-range atmospheric transport (LRAT). The deposition of dioxins in soil is heavily influenced by global air currents and local weather patterns that transport these compounds from the industrialized regions responsible for over 80% of global emissions to less industrialized areas. Consequently, dioxins can be deposited in soils across both industrialized and developing countries, with up to 57% of global dioxin emissions eventually settling in the soil [30]. In regions where dioxins are deposited, soil can serve as a long-term reservoir for these persistent and toxic compounds. Research indicates that soils in areas affected by significant LRAT can have dioxin concentrations like those found in locations with active industrial emissions. For example, industrial activities, the use of outdated pesticides, and household heating contribute to the PCDD/Fs accumulation in the soil, often at levels comparable to those in developed countries [43].

This shows the widespread occurrence of dioxins across different environments, with soil acting as a major source of their persistence. Generally, a high concentration of dioxin is recorded near waste handling and industrial areas. The urban area shows a moderate level of dioxin, and undeveloped and rural areas show low concentrations, although deviations from this pattern can be caused by LRAT. Several regions around the globe face significant dioxin contamination, with varying sources and concentrations, as shown in Table 2.

This development shows how the soil contamination level is highly affected by different strategies such as industrial activities, ecological management, and regional policies. In one example, reference [56] shows that higher concentrations of dioxin are typically found in soil near the production zone, including in urban, mountainous, and agricultural regions. A study by Liu et al. [57] observed that dioxin concentrations were notably higher in soils near industrial and agricultural sites, with levels varying depending on the distance from pollution sources and local environmental conditions [57]. Research indicates that dioxin levels in soils [52] predominantly originate from incineration processes and agricultural activities, although there are fewer recent studies available on the topic [53].

Table 2 further shows that dioxin levels are different worldwide, which is influenced by several factors, as discussed earlier, like local industrial areas, practices of waste management, and different sources of combustion. The dioxin level is recorded as being high near the electronic waste sites and industrial areas, while moderate levels are found in urban regions and lower levels are found in rural or less industrialized areas. However, the LRAT leads to a rise in the dioxin level even in the regions with low local emissions, which proves that contamination is not limited to emissions. These findings show that both local and long-range sources must be considered when assessing the contamination risk in soil [58]. Dioxin levels in Europe have not declined in recent years; instead, they have risen substantially, influenced by developing sources like the improper disposal of electrical transformers, as shown in [52,59]. As shown in [45], apart from industrial emissions, motor vehicle releases also contribute to pollution because of the additives used in fuel combustion.

A comparison of dioxin levels across various European regions reveals that a few countries exhibit lower soil dioxin concentrations compared to others. Studies on the levels of dioxin associated with sources like incineration of municipal and clinical waste and industrial emissions report concentrations ranging from 0.45 to 14.41 ppt-TEQ (dry weight), with uncontrolled burning procedures being the most impactful contributor. Research conducted in the UK highlights that dioxin levels tend to be high in urban soils, as compared to rural areas, due to uncontrolled combustion [9].

Like the findings from research documents on dioxin contamination in the soil samples of different countries in Europe, there is limited data on soil research in recent years in America. Generally, the concentrations of dioxin in urban soils across the USA are typically higher than those in rural soils, with urban areas showing maximum concentrations of 186 ppt-TEQ. A study done in Washington reported a comparatively low level of dioxin in soils, with a range of 0.14 to 4.1 ppt-TEQ. The lowest concentrations were observed in agricultural land and the highest in urban land, consistent with findings from other research [9]. Data on dioxin levels in soils across African countries are scarce because of the high analysis cost and limited technical capacity. Research by Nieuwoudt et al. [60] indicates that concentrations of dioxin in soils range from 0.34 to 20 ppt-TEQ, which are lower compared to those reported in Europe and the United States. The study revealed that dioxin levels were highest at industrial sites, followed by agricultural soils, and were lowest at non-industrial sites. Different combustion sources were identified as the main contributors to pollution. Additionally, power generation and specific petrochemical operations may also contribute to the release of these pollutants [60].

The inconsistency of dioxin pollution in regions of the world underscores the necessity of region-specific remediation measures. Nevertheless, the similar aspect of all the sites that are contaminated is the need to conduct a systematic study that focuses on treatments depending on the levels of contaminants, the characteristics of the soil, and the possible routes of exposure. A comprehensive remediation model has the ability to respond to these regional disparities and adhere to fundamental sustainability and effectiveness principles.

## 3. Soil Contamination by Dioxins

Dioxin-like chemicals (DLC), including DL-PCBs, PCDD/Fs, and other similar PAHs, are very stable and can accumulate in the water, living organisms, the soil, and the ecosystem. They enter the food chain and badly affect both wildlife and humans, as well as cause contamination in the environment. Dioxin exposure can cause severe health issues like cancer, reproductive system problems, skin problems, a weak immune system, and other toxic effects [39]. Dioxins stored in these “reservoirs” can be dispersed again and moved through processes such as the resuspension of sediments or dust transport [7]. Photochemical procedures, including photolytic radical reactions, occur when pentachlorophenol is exposed to ultraviolet (UV) light. The UV radiation breaks the chemical bonds in pentachlorophenol, generating reactive radicals that can then recombine to form dioxins. Different accidental causes of dioxin release, including major incidents like the Seveso disaster in Italy and the Yusho poisoning in Japan, where large volumes of dioxins were accidentally discharged into the atmosphere, resulted in widespread contamination. Furthermore, the industrial accidents, natural events like fires in the forests, and volcanic eruptions can contribute to the release of dioxins as well, as the high temperatures during these events can lead to the combustion of organic material, leading to the formation and release of these harmful compounds [58]. The various sources of dioxin formation include different environmental and industrial processes such as the operation of fluidized bed combustion boilers (FBC), thermal oxygen cutting of scrap metal at destruction sites, house fires involving polyvinyl chloride (PVC), tire incinerators, Kraft liquor boilers, power generation, disposal of laboratory waste, barrel and drum reclaiming, reactivation of carbon furnaces, and recovery of scrap electric wires [61].

**Figure 4:**
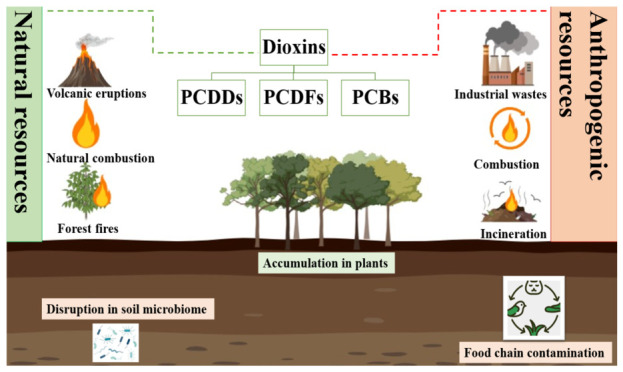
Source and impacts of dioxins in the soil. Dioxins in soil originate from natural and anthropogenic activities. They disrupt plants and soil microbiomes and enter the food chain. BioRender was used for figure creation. BioRender: Scientific Figure and Illustration Software.

Dioxin pollution in soil is a growing environmental concern. Dioxins are extremely toxic environmental pollutants, posing high health risks to humans and a threat to the environment. Their toxicity results from their long persistence in the environment. They can persist in the soil for decades because of their structural stability. Accumulation of dioxins in the soil can be both anthropogenic and natural, as shown in Figure 4. The natural sources of dioxins are forest fires [62], natural combustion, and volcanic eruptions. Anthropogenic sources of dioxin accumulation are waste incineration, tobacco smoke, combustion, fly ash, and waste from chemical industries [63].

Soil is considered a main sink for the accumulation of dioxins because of their lower solubility in water [10]. Vegetation on the surface of the soil can become directly contaminated with dioxins when airborne particles settle on leaves, often carried by atmospheric currents. Additionally, plants can absorb dioxins through their root systems, which transport these toxins from contaminated soil into the plant tissues [64]. Extensive research has been done on the effect of dioxins, particularly TCDD, on the growth of terrestrial plants. *Arabidopsis thaliana* exposed to TCDD exhibits reduced seed germination, variation in transcription factor genes such as *AP2-EREBP*, *MYB*, and *bHLH*, and decreased fresh weight and altered root structure [65]. One study done by Hanano et al. [66] demonstrated the accumulation of TCDD in vegetative plant tissues, especially in mature seeds and rosette leaves, which led to delayed flowering and seed yield with low vitality and lower oil content [66].

Dioxins are highly available to soil microorganisms because of their lower solubility in water and higher affinity for soil organic molecules. The bioavailability of dioxins to microorganisms varies among several species. Some bacterial species can form surface-active molecules such as biosurfactants. Biosurfactants reduce surface tension among organic molecules and bacteria [67]. Several studies have been done to control the toxicity of dioxins in bacteria. Soil microorganisms exposed to dioxins experience various biological responses that impact their functionality and diversity. The dioxins are highly lipophilic, which means they have a high affinity towards lipids, making them highly toxic. Dioxins particularly influence lipid metabolism in soil microbes, altering cell membrane composition [10]. However, dioxins can penetrate the food chain through soil contamination, as demonstrated by a veterinary inspection-based study in Poland. They found high levels of dioxin in eggs laid by hens foraging in contaminated soil [68]. In another study, the dioxin levels in tissues of animals living close to landfills were found to be 38 times higher than the Russian set standard [69].

### Priority Pollutants in Soil

**Figure 5:**
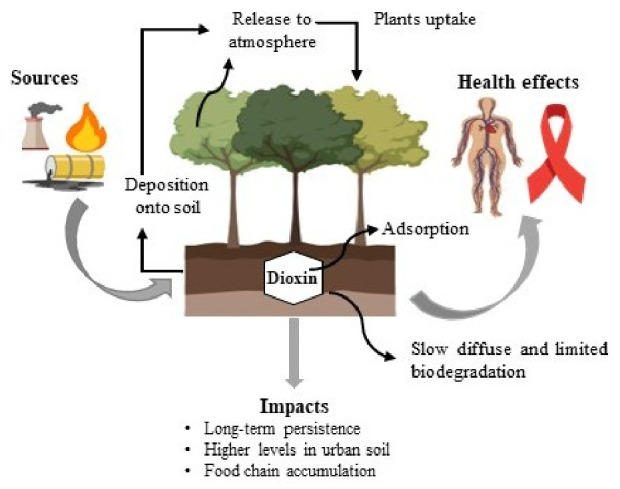
Dioxin as a persistent pollutant in soil. BioRender was used for figure creation. BioRender: Scientific Figure and Illustration Software.

Dioxins are considered the most persistent and toxic organic pollutants in the soil, with ecological half-lives reaching up to a century. Due to their severe health and environmental hazards, dioxins are classified as a priority pollutant. Urban and suburban soils mostly show dioxin levels between 0.1 and 186 ng/kg TEQ, a measurement representing the total toxic effect of dioxins that generally exceeds those found in rural areas, which range from 0.1 to 22.9 ng/kg TEQ [13]. Industrial discharge contributes most to soil dioxin contamination, followed by the residual effect of military activities, and combustion sources. These contaminations cause high levels of health risks and the contamination accumulates in the food chain over time, as shown in Figure 5. Ecologically sustainable remediation techniques including phytoremediation, bioaugmentation, and biostimulation are normally used to address soil contaminated with dioxin. Soil microbial communities in the environment often adapt by upregulating dioxygenase gene expression, mostly in highly polluted areas. Besides these advances, managing emissions of gases and chlorine-based radicals during the treatment remains a major challenge, underscoring the importance of continued investigation [4]. Dioxin contamination in soil poses a high risk of toxic effects on human health and the environment. Globally, soil dioxin levels arise from different causes, with the primary contributors being the remainders of conflicts and human activities. The European Union (EU) has set the maximum limits for dioxin contamination in food products. Mostly, the maximum acceptable level is 2.5 pg PCDD/F-TEQ/g of fat for PCDD/Fs alone, whereas 5 pg PCDD/F-PCB-TEQ/g of fat applies to the concentration of PCDD/Fs collectively with DL-PCBs [70]. The European food safety authority (EFSA) has recognized 2 pg TEQ/kg body weight/week of dioxin as a tolerable weekly intake (TWI). Furthermore, the average exposure of adults, approximately 1.8 pg TEQ/kg bodyweight per week, is considered to be below the recommended limits; however, children’s exposure to dioxin or DLCs, estimated at 3.4 pg TEQ/kg bodyweight per week, exceeds the TWI. This lead to a high health risk for the younger population and should be highlighted in the risk characterization zone [71]. According to a report by the World Health Organization (WHO), the safe daily exposure to dioxin should range from 1–4 picograms per kilogram of body weight or should be 0.07 ppt in blood. In most countries, the standard level of dioxin in the environment is set at 150 ppt in sediments and 1200 ppt in soil. The United States Environmental Protection Agency (EPA) has suggested keeping the level at 72 ppt-TEQ to allow for the safe treatment of larger amounts of contaminated soil. Dioxin has a life cycle ranging from 60 to 80 years in soil and persists in the ecosystem for a long time, infiltrating sediments and soil and moving into plants and aquatic organisms. This results in the accumulation of dioxin in both the soil and throughout the food chain [20].

Environmental pollution, especially dioxin contamination in soil, is significantly increasing. Dioxin emissions from industrial countries contribute to over 80% of the assessed annual dioxin emissions. In 2015, Dopico and Gómez [72] stated that major contributors to dioxin contamination in soil include metal production, pesticide manufacturing, fuel combustion, landfill disposal, waste incineration, combustion processes, accidental fires, and herbicide runoff from agricultural activities [72].

According to Booth et al. [30], the annual production of dioxins is approximately 17,226 kg (287 kg TEQ) globally, with around 57% (163 kg-TEQ) settling on soil, 40% (115 kg TEQ) in ocean waters, and 3% (9 kg-TEQ) remaining in the air. As the soil acts as the major reservoir for dioxins, which contributes to their long-term stability in the environment and their accumulation in food chains, it poses a threat to both ecological systems and the human population by causing different serious diseases [30]. Soil-deposited dioxin can be taken up by plants, which are then utilized for the consumption purposes of animals, leading dioxins to enter the food chain. The contamination of soil plays a major role in the spread of dioxins within the environment. Plants growing in polluted soils contribute significantly to the transfer of dioxins to animals, and finally to the human population by consumption. Soil has been declared to serve as a critical environmental reservoir for the release of dioxin from multiple emission sources.

Dioxins, including PCDDs and PCDFs, are considered the most hazardous class of POPs. The compounds are made up of two aromatic rings, which are joined either by one or two oxygen atoms, resulting in the formation of PCDFs and PCDDs, respectively. Their structure allows for the attachment of up to eight chlorine atoms, which contributes to their high hydrophobicity and strong chemical stability. Based on the number and chlorine atom locations, which range from 1–8, there are 135 PCDFs and 75 PCDDs variants, each with various levels of toxicity. The variants with chlorines at positions 2, 3, 7, and 8 are considered the most toxic, with the (TEF) toxic equivalency factor of 1.0 [73].

As priority pollutants, dioxins require remediation strategies that are not merely remediation strategies, but risk reduction strategies that involve recovery of the ecosystem in the long term. The single techniques that are traditionally employed have been found to be inadequate because dioxins are complex in their behavior in soil. Thus, a staged perspective that incorporates the preventive approach to risk, followed by the biological recovery of the soil, is a more feasible road to soil health.

## 4. Dioxins’ Effects on Human Health and the Food Chain

Dioxin is the most common highly toxic pollutant released into the environment through anthropogenic activities and has a very severe effect on animals and human health. Soil contaminated with dioxin can introduce dioxin into the food chain and lead to human exposure, mainly through the food chain, as the dioxin can accumulate in animal fats as well. The ingestion of contaminated soil, directly or by contact with the skin, can occur, but is usually less significant than consuming dairy, meat, or fish contaminated with dioxin [74].

**Figure 6:**
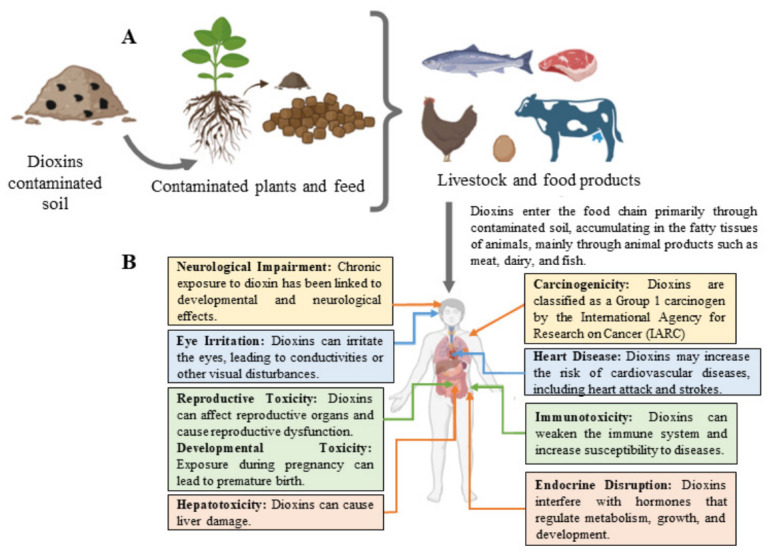
Dioxins transfer through the food chain and have an effect on human health. (**A**) The sources of dioxins in the food chain and (**B**) the health impacts caused by dioxins on humans. BioRender was used for figure creation. BioRender: Scientific Figure and Illustration Software.

PCDDs, PCDFs, and PCBs are organic pollutants that have severe effects on human health. Once they are released into the atmosphere, they adhere to particulate matter. Via atmospheric deposition, they settle onto soil and vegetation, successively contaminating crops such as cereals, vegetables, and fruits. As per the statistical data, 90% of dioxin intake is via ingestion. The food chain is the key cause of dioxin ingestion in humans, as shown in Figure 6A. The contamination is found in the fatty tissues of fish and animals and the eggs of poultry fed on contaminated feed. The direct-entrance pathway of PCDD/Fs to the human body is through contaminated cereals and vegetables, while the indirect pathway is through livestock, as cattle ingest contaminated grass and soil; consequently, humans are exposed to PCDD/Fs by consuming contaminated eggs, milk, meat, fish, and dairy products [75].

Dioxins are persistent endocrine-disrupting chemicals. Their toxicity has been well-documented in mammals. This is influenced by factors like exposure concentration, duration, and exposure route [76]. Dioxins can cause cancer in organs like the skin, thyroid, liver, and lymphatic system [77]; they also contribute to breast cancer [78]. They also contribute to immunosuppression, teratogenicity, metabolic disruptions, and dermal issues such as chloracne and hair loss, as shown in Figure 6B. 2,3,7,8-TCDD is a notorious compound of the dioxin family because of its toxic impact on human health. TCDD is well-studied for its role in harming human metabolism. The short-term effects of exposure to TCDD include alteration in the functioning of the liver and skin irritations, including skin darkening, and chloracne. The human health impacts of long-term TCDD exposure include soft tissue sarcoma, digestive system cancer, non-Hodgkin lymphoma, multiple myeloma, malignant neoplasms, and lung cancer [75]. Long-term exposure to TCDD can cause cardiovascular disease through several mechanisms, including atherosclerotic plaque, meaning the buildup of fats in the blood vessels that blocks and narrows the arteries and disrupts blood circulation [79].

Furthermore, dioxin has been linked to diabetes and thyroid disorders, as well as other respiratory issues, such as bronchitis and lung function. Additional effects include altered serum testosterone levels, gum and eyelid pigmentation, vomiting, loss of appetite, and severe skin conditions such as rashes. Other findings included increased hair growth and dental anomalies, such as enamel hypomineralization of permanent first molars, in children [80]. Table 3 explains the dioxins and their toxic effects on human health.

Dioxins also cause liver dysfunction by causing hepatic steatosis. This condition is characterized by the extreme buildup of fat in liver cells. That causes dysfunction of the liver. TCDD can also suppress the activation of antioxidant enzymes in the liver, which ultimately causes oxidative stress in the liver [86]. Dioxins can modify various pathways in the nervous system and can cause complete neuron impairment. They can stimulate reactive oxygen species (ROS) generation and cause oxidative stress. The chemical also has destructive impacts on tissues of the brain, in particular, causing neuronal apoptosis, secondary brain damage, and neurological defects [87]. Another study reported that even a small quantity of TCDD (approximately 10^−10^ mol/L) can increase the expression of the neurofilament light chain (NFL) [88]. NFL is a subunit of neurofilament (NF), which is an important neuron component. Overexpression of NFL can cause accumulation of NF at abnormal levels, which ultimately causes neurodegeneration [89]. Research has shown that TCDD exposure can affect energy metabolism by disturbing fat and glucose metabolism in humans. Its exposure leads to glucose intolerance in mice. Further studies showed that if mice are fed a high-fat diet, TCDD exposure can lead to hepatobiliary injury, cardiac arrest, and neuron impairment [79].

## 5. Toxicity Mechanism

Dioxin and its congeners can cause toxicity through various molecular mechanisms in both wildlife and humans when they are exposed and come into contact with mixtures of dioxin compounds. Several molecular mechanisms have been identified that explain how dioxin compounds can cause toxic effects, including interference with gene expression and disrupting cellular, biochemical, and tissue differentiation processes [7,90]. The most well-known mechanism by which dioxin exerts toxicity is its activation of the AhR signaling pathway, which results in a variety of biological effects [7].

In this canonical pathway, lipophilic agonists such as (TCDD) 2,3,7,8-tetraclorodibenzo-p-dioxin enter the plasma membrane, where they bind to the AhR receptor. The binding causes the release of chaperone proteins and the formation of an AhR agonist complex, which then binds to the AhR nuclear translocator (Arnt). This complex is transported into the nucleus, where it attaches to the specific sequences of DNA known as xenobiotic responsive elements (XRE) located in the promoter sequences of different genes. This binding activates the gene transcription, such as cytochrome P450 (*UGT1A1*, *CYP1B1*, and *CYP1A1*), the AhR repressor (*AhRR*), and various enzymes that downregulate AhR signaling [91]. Luciferase transcription is often measured in a reporter gene to measure the activity of this pathway; in this approach, luciferase enzyme production is proportional to the amount of light produced, correlating to the concentration of protein in the exposed cells [92,93].

**Figure 7:**
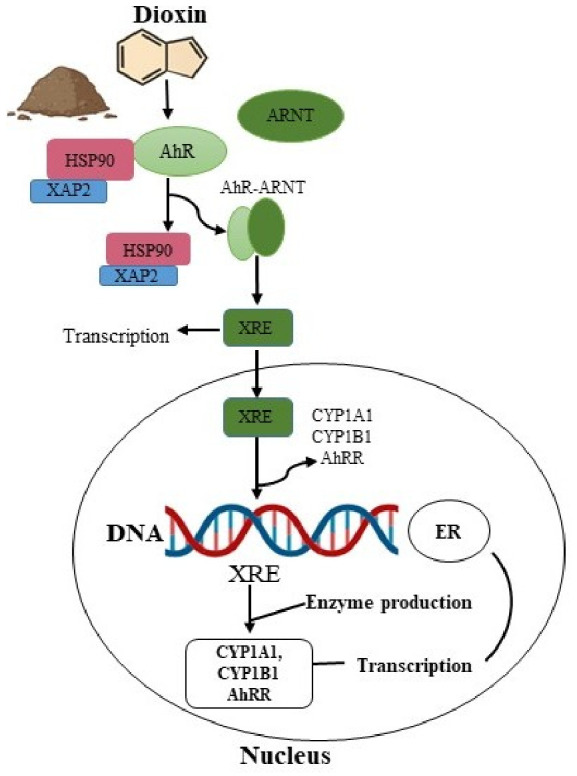
Mechanism of dioxin-induced toxicity in soil. Biorender was used for figure creation. BioRender was used for figure creation. BioRender: Scientific Figure and Illustration Software.

Apart from the canonical pathway, AhR also engages with various other receptor-mediated signaling pathways. AhR activation can disrupt endocrine signaling, including that mediated by steroid hormones. Depending on the environmental condition, ligand, tissue, and cell type, activation of AhR can regulate the cell cycle, activate mitogen-activated protein kinase cascades, induce immediate early genes, and crosstalk with estrogen receptors (ER) or additional nuclear receptors [94,95], as illustrated in Figure 7. The AhR–Arnt complex has been shown to activate estrogen receptor-dependent genes, or in their absence, interact with estrogen receptor (ER) dependent genes by binding to them, even without an estrogen agonist present [96].

Dioxins bind to the AhR protein, which undergoes a conformational change upon binding. This AhR division into two complexes affects the function of nuclear steroid receptors, disrupting their activity through two primary mechanisms. First, dioxins can increase the activity of nuclear steroid receptor-dependent genes like estrogen-responsive elements (ERE) by forming a transcriptionally active complex that includes AhR and Arnt coactivators (e.g., p300), as well as estrogen receptors. This complex stimulates the transcription of ERα genes even in their absence, while the second mechanism involves the formation of AhR–Arnt–ERα complexes that reduce the response of ERα to estrogen, leading to disruption in the transcription of steroid-dependent genes in estrogen-responsive tissues [97].

Furthermore, dioxins, through the action of proteasome, can modify the level of steroid receptors, including ERα, through degradation by the ubiquitin ligase complex (CUL4B-AhR), which is specific to the nuclear steroid receptor group member, for example, ERα [94]. This complex forms after the interaction of the dioxin and the amino terminus of CUL4B, triggering the degradation of ERα by ubiquitination. Studies have shown that the reduction of any component of this complex decreases the ubiquitin chains on ERα and prevents the degradation of ERα dependent on dioxin [97]. This mechanism plays a role in dioxin’s function as an endocrine disruptor, affecting hormone receptor function [94]. The occurrence of ERα and AR in high concentrations in reproductive tissue exposed to dioxins suggests that these receptors may be unresponsive to dioxins in the absence of AhR. Furthermore, cells with normal or absent AhR, when depleted of CLU4B, fail to suppress hormone-dependent gene expression and cell proliferation. Moreover, AhR may modulate the immune system by promoting the expansion of Treg cells, activating the transcription factor NF-KB, and modulating the balance between IL-17 and IL-22 production [95,98]. AhR agonists can either mitigate autoimmune diseases or promote inflammation, depending on the context. Interestingly, natural AhR agonists, which are present in vegetables, typically do not cause adverse effects such as chloracne, but might cause a chloracne-like condition without AhR activation [94].

The complex mixtures of DLC complicate the evaluation of the health risks to the human population. Estimating the concentration of each congener does not always reflect the full extent of AhR activation or signaling pathways [99]. TEF is used to assess the overall toxicity of dioxin and its congeners by considering their ability to activate AhR [100,101]. TCDD is typically given a TEF value of 1.0, and the total toxicity of dioxin mixtures is determined by multiplying the concentration of each congener by its respective TEF, resulting in the dioxin TEQ. The TEF concept simplifies the assessment of health risks by reducing uncertainties in human health risk assessments. However, the TEF method is very time-consuming and expensive, requiring high sample volumes and the use of gas chromatography mass spectrometry [92].

TEF is linked with critical limitations. WHO reported that TEFs present an order of magnitude of uncertainty, based on various relative effect potencies (REPs) from certain tests. Most of the TEFs originate from rodent intake data, whereas the REPs are measured in tissues or blood vessels and can differ mostly from intake values, leading to risk assessment errors. They are mostly rodent-derived, not human; their major data is derived from rodents. TEFs from rodents can also be applied to humans, even given the differences in species; for instance, humans are about 10–100 times less sensitive, compared to rodents, in terms of PCB 126. The WHO-TEF is also rodent-based data [102].

There is also the assumption of the TEF approaches that dioxin-like toxic effects can occur through the AhR only, overlooking the interaction of compounds and possible non-AhR mechanisms. These assumptions may increase the effects at high doses and exclude the chemicals that compete with the AhR or partially activate it. Furthermore, the procedure depends on the REP data that shows high uncertainty and variability, mainly in the interspecies extrapolation. The TEF approach has limitations, assuming that all “dioxin-like” toxic effects occur only through the AhR. This neglects the other toxicity pathways, which possibly causes the overestimation of effects at high concentrations and fails to account for the compounds that act as incomplete agonists or competitors for AhR [103]. The conclusion from such a limitation proves that the TEF model is basic and should be carefully used in risk assessment. Despite these drawbacks, TEFs are crucial for providing retrospective and prospective assessments of the possible health risks that are posed by dioxin exposure [101].

The toxicity of dioxins mediated by AhR underscores that the reduction in biologically available fractions, but not concentrations, should be regarded as the primary goal of mitigating soil pollution. Remediation plans should thus seek to reduce bioavailability by degrading or sequestering. A combined system whereby the mobilization and detoxification of dioxins occur in a different sequence, utilizing physical, chemical, and biological techniques, can be efficient in attacking the toxic pathways of dioxin.

## 6. Remediation Strategies for Dioxin-Contaminated Soils

**Figure 8:**
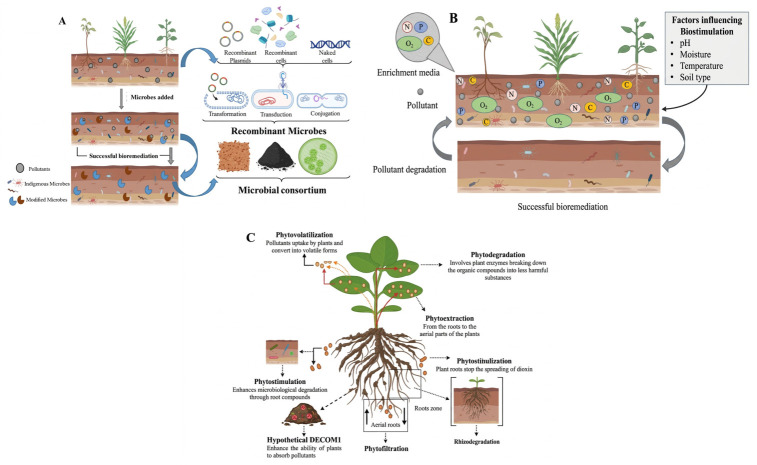
Bioremediation techniques used for the treatment of dioxin-contaminated soil. (**A**) Bioaugmentation involves specific microbial strains, which are used to improve the dioxin degradation. (**B**) The biostimulation procedure promotes the indigenous microorganism’s activity by using nutrients. (**C**) Phytoremediation utilizes plants to facilitate the pollutant degradation and uptake. BioRender was used for figure creation. BioRender: Scientific Figure and Illustration Software.

Dioxin levels at contaminated sites often exceed the regulatory limits set in environmental guidelines. As a result, considerable efforts have been dedicated to cleaning up these areas. Among the various remediation approaches, soil treatments have gained attention. Various techniques for treating dioxin-contaminated soil have been utilized: chemical and physical remediation (conventional and widely used) and biological techniques (eco-friendly), as indicated in Figure 8.

### 6.1. Biological Remediation Techniques

Bioavailability refers to how easily living organisms, including plants and microbes, can absorb or degrade contaminants. The significant factor determines the effectiveness of biological remediation. The exposure risk of dioxin compounds in the soil is significantly influenced by it. The procedures, such as soil aging and accumulation of organic carbon compounds, such as black carbon, lead to the bioavailability of hydrophobic compounds like PCDD/Fs. The strong bond and interaction of aged dioxin compounds with soil organic matter is one of the major problems in their treatment. This interaction limits the availability of dioxin, and the efficacy of the remediation procedures is reduced. The dioxin is kept trapped in the soil structure by such a strong bond, which makes them less available for uptake by plants or degradation by microorganisms [104]. Methods of biological treatment are widely regarded as effective and eco-friendly approaches for reducing pollutants in various environments. One such method, bioaugmentation, introduces the specific microbes into the soil to improve the breakdown of the contaminants, proving it to be a promising strategy for bioremediating contaminated soil with organic pollutants [105]. The processes where the microbial activities can be enhanced by adding different nutrients or modifying the soil nutrients are referred to as biostimulation [106].

Composting is another process used for the conversion or breaking down of organic waste into the simplest organic compound. The process has been regarded as an environmentally friendly approach for addressing organic soil contamination, such as that involving dioxin, furans, or petroleum. The processes of composting are further divided into several phases, such as mesophilic, cooling, and thermophilic stages, which are determined by different activities of microorganisms and the associated heat generation [107]. The mesophilic phase, occurring at temperatures below 45 °C, is characterized by the rapid adaptation and growth of microbial populations, as they utilize easily degradable organic materials [108]. Another mechanism, phytoremediation, is often explored for its ability to restore soil contaminated with persistent organic compounds, enabling its potential reuse [24]. Phytoremediation mainly relies on mechanisms such as absorption by plants and the accumulation of harmful substances [109].

#### 6.1.1. Bioaugmentation

Generally, bioremediation is the use of different plants, or their enzymes and microbes, for the removal of environmental pollutants. These techniques include biostimulation, which involves adding nutrients to enhance the native microbes, and bioaugmentation, which introduces specific microbes to improve cleanup processes. Bioaugmentation is a bioremediation approach in which microbes with specific pollutant-degrading capabilities are introduced into contaminated environments such as water, soil, or sediments to enhance the breakdown of the hazardous substances [105]. Certain microbes, especially bacteria, can survive in the toxic environment by developing mechanisms of adaptation that help them combat contaminants. These adaptations arise through natural selection, where ecological pressure drives changes in their phenotype and genotype, allowing them to persist under harsh conditions. Bacteria protect themselves in a polluted environment by developing resistance to toxic substances like different heavy metals, allowing them to survive and maintain their population. These resistance strains can tolerate and biologically eliminate harmful metals through processes including bioreduction or oxidation, biotransformation, bioaccumulation, and biosorption [110].

Bioaugmentation enhances the treatment performance by introducing the selected strains into a biological system, which can lead to notable improvement in contaminant elimination and restoration of microbial community function. Advances in high-throughput sequencing and metagenomics have greatly improved the ability to analyze complex microbial populations and metabolic pathways in detail. These technologies now offer valuable insights into the underlying mechanism of bioaugmentation [111]. Bioaugmentation deals with the use of either naturally occurring or genetically modified microorganisms in contaminated soil to accelerate the removal and breakdown of hazardous heavy metals from the soil. Figure 8A provides a visual representation of bioaugmentation. Bioaugmentation is commonly applied to ecological pollutants with heavy metals and petroleum contaminants. Different microbial formulations are used for the hydrocarbons’ bioremediation, such as strains including *Rhodococcus* and *Pseudomonas*. Various hydrocarbon-degrading bacteria have been identified, such as *Stenotrophomonas*, *Microbacterium*, *Gordonia*, *Dietzia*, *Ochrobactrum*, *Brevibacterium*, and *Aeromicrobium* [112].

Bioaugmentation contributes to broadening the gene pool and enhancing genetic diversity at the site of the contaminated soil. Increasing the wide range of different microorganisms can greatly increase genetic differences. A critical aspect of successful bioremediation is the capability of the introduced microbial consortia to adapt to the specific conditions of the contaminated soil. Furthermore, their success depends on how effectively they can compete with native microbial communities, resist predation, and withstand different abiotic stressors. This process enhances the efficacy of the remediation processes by improving the capacity of the soil [113].

Consortia bioaugmentation is the technique where a community of different microorganisms with the ability to degrade different pollutants is used. In a cold ecological system, the different biotic factors, including the interspecies competition among the exogenous and indigenous microbes, and abiotic factors like moisture, organic matter, pH, nutrient contents, characteristics of contaminants, and aerations, must be measured in the selection of strains that are suitable for degradation purposes. Both psychrotolerant bacteria, which can tolerate temperatures of more than 25 °C, and psychrophiles, which can grow at or below 15 °C, can degrade dioxin-like pollutants in cold environments [114]. Native microbes are far better than inoculants in the cold environment. The most effective bioaugmentation depends on the distribution and survival of the microbes introduced. The use of suitable inoculation techniques with carriers as biosolids, charcoals, manure, peat, or clay, and suitable other materials that are immobilized, such as gelatin, alginate, and agarose, are important; all these improve the survival rate by providing protection and a microenvironment, as mentioned in Figure 8A [115].

Recombinant bioaugmentation is the procedure used in the degradation of pollutants in the soil. In these techniques, the genetically modified microbes (GMMs) are introduced into the soil, along with the free remediation genes or plasmid carrier genes. This procedure has the capability for the transfer of the prevalent degradative genes from one ecosystem with a cold temperature to another cold ecosystem. The technology allows for operon or gene modifications and catabolic construction, as well as changes in the existing catabolic genes [116]. *Pseudoalteromonas haloplanktis* TAC125, a psychrophilic bacterium, was genetically modified with biodegradative genes encoding toluene-o-xylene monooxygenase (ToMO), which was achieved from *Pseudomonas stutzeri* OX1, known as a mesophilic strain. The genetically engineered *Pseudomonas* showed the ability to degrade 2,4-dinitrotoluene, even at low temperatures. In addition to the degradation of pollutants in soil, the recombinant bacterial strains are used for different purposes, such as strain monitoring, endpoint analysis, measurement of stress response, and assessment of toxicity [117].

The survival of recombinant bacterial strains depends on many factors in the soil, such as the existence of competitors and predators, various abiotic factors, the density of inoculum, and soil microflora. Additionally, temperature plays a key role in the metabolic and molecular activities of the recombinant bacteria. The GMMs used for bioremediations have limited applications because of the genetic material instability in the soil. Regarding the limitations, directly introducing the self-transmissible plasmids or naked remediation genes into the soil for degradation purposes is beneficial. The horizontal gene transfer enables the catabolic gene incorporation into indigenous microbial strains, as mentioned in Figure 8A. Naturally occurring horizontal gene transfer plays a key role in enabling native microorganisms to acquire the ability to degrade [118]. This approach is very advantageous in cold areas, where the survival of non-native microbes restricts the degradation capability [117].

Microorganisms can be utilized for different purposes, but the use of different microorganisms for treating dioxin-contaminated soil is a growing approach currently because it is cost-effective and has a very eco-friendly nature. Biological degradation of dioxins primarily occurs through microbial dechlorination, which can take place under two different conditions: anaerobic and aerobic [119]. Microbial strains can use dioxins as a source of carbon and energy, enabling the effective dechlorination of highly chlorinated isomers [120]. Table 4 shows that different microbial strains are employed for dioxin degradation. Specific microorganisms, including *Pseudomonas*, *Dehalococcoides*, and *Mendocino*, have efficiently proved their ability in the dechlorination of dioxins in anaerobic environments [121,122].

Moreover, aerobic microorganisms have been found to degrade dioxins more rapidly and efficiently, in comparison to anaerobes commonly present in dioxin-contaminated soils. Among aerobic degraders, *Bacillus firmicutes* is the predominant strain [130]. The microbial strains that efficiently degrade dioxins and fungi also contribute significantly, with their large biomass and fast metabolism in the environment. Fungi are highly varied organisms found in various environments, where they play essential roles in these environments. They can regulate the energy and flow of nutrients within their networks [108].

Fungi are distinctive organisms that can be utilized for the remediation purposes of different POPs in various environments, including water, air, and soil. Dioxin-contaminated soil can benefit from fungal decomposition, with certain strains demonstrating high efficiency. Notable fungal strains, including *Phanerochaete sordida* YK-264, *Phlebia lindtneri*, and *Cordyceps sinensis* strain A, have shown effectiveness in breaking down dioxins. Table 4 lists additional fungal strains that are used for dioxin decomposition in soil [9]. Ishii and Furuichi [127] proposed a bioreactor system using the fungus *Pseudallescheria boydii* for the treatment of soil contaminated with 2,3,7,8-TCDD. Ideal conditions for the fungal activities were identified, such as a chloride level below 10 g/L and a pH below 9. Under these parameters, the level of dioxin was reduced significantly, and a post-treatment of ethanol extraction improved the removal efficiency to 92%. The treatment was conducted in an A5-L stainless steel cylindrical reactor equipped with an angled turbine operating at 200 rpm, which was optimized through preliminary tests.

Overall, bioaugmentation has shown improvement in the bioremediation process, but there are several challenges associated with it. These include microbial survival, competition with indigenous communities, environmental variability, and the impractical separation of the metals from the soil. These limitations are faced by bioaugmentation in real-world applications [131]. Other limitations involve high levels of chemical and energy consumption for the separation of electrochemistry, as well as the requirement of refined laboratory equipment, such as magnetic and electric separators. There is also metal-releasing potential because of the changes in redox conditions and pH [132].

However, advances in molecular biology have enabled the development of strategies to enhance microbial robustness and to design synergistic consortia tailored for the degradation of complex contaminants. Strong resistance and multifunctionality are shown by microbial consortia, in which different bacterial species cooperate to use the available substances in the best way. This combined effort makes them more effective in bioremediation in comparison to using single microbes. The recent progress, such as the development of resistant microbial consortia, molecular tools, and genetically modified microorganisms, offers modern ways to address such limitations. The effectiveness of the bioaugmentation procedure for the remediation of different pollutants from the soil can be improved by these strategies, even in unfavorable environments [133].

#### 6.1.2. Biostimulation

Microorganisms native to lead-contaminated environments play an important role in the ecological cycling of metals and have important potential as bioremediation agents. Biostimulation also aims to increase the activity of indigenous microbes capable of breaking down the pollutants by altering the ecological conditions. This is mainly achieved by supplying the important nutrients, as well as electron acceptors such as nitrogen, phosphorus, oxygen, or carbon sources, which are considered important for microbial activity but may be available in low amounts, as mentioned in Figure 8B. This can help in restoring the polluted sites and reducing the toxic impacts of different heavy metals in soil. Additionally, signaling molecules, including hydrogen sulfide and nitrogen oxides, when applied externally, mainly in the legume rhizobium symbiotic system, can suppress the uptake and accumulation of pollutants in plants. These molecules support detoxification by reshaping the microbial community structure and enhancing the enzymatic functions within the contaminated soil [134].

Several factors can restrict the biodegradation processes in soil, such as deficiencies of nutrients, pH, soil type, level of moisture, availability of oxygen, temperature, the existence of contaminants, and the characteristics of the specific soil [135]. Microbial adsorption plays an important role in the transformation and breakdown of organic contaminants during the process of composting. Additionally, various operational factors like environmental conditions, humidity, and the ratio of carbon–nitrogen (C/N) are essential in the process of bioincubation. Humidity is the main factor influencing the degradation processes of different organic contaminants effected by microorganisms, affecting both the microbial activities and the various physicochemical properties of the contaminants [136].

Oxygen molecules are involved in the mineralization and catabolism of hydrocarbon compounds through the activities of microorganisms. Furthermore, the carbon and nitrogen ratio significantly influences the biodegradation of the targeted contaminants by regulating the microbial community’s composition. Previous research shows that the ideal carbon–nitrogen ratio for the process of biodegradation of organic contaminants through bioincubation ranges from 10 to 40. Bio-composting has also proven effective in the process of biodegrading soils contaminated with dioxin in laboratory studies [137]. Chen et al. [23] reported that the biodegradation efficacy of PCDD ranged from about 95.8% to 99.7%, reducing the primary toxic level of 1580–3660 µg I-TEQ/kg dry weight after the incubation period of 42 days [23]. Table 5 provides a summary of the organic materials used for dioxin degradation in soil through biological composting.

A study was completed by Huang et al. [139] in which highly PCDD/F-contaminated soil was investigated by using aerobic food waste co-composting; this was followed by monitored natural attenuation (MNA). The treatment was carried out in a 0.2 m^3^ bench-scale composting reactor equipped with rotation control, air injection, and sampling channels. The co-composting processes were maintained for 28 days, after which MNA continued until day 42. The initial concentration of dioxins, 16,004 ng-TEQ/kg, was reduced to 4001 ng-TEQ/kg, achieving almost 75% removal. Next-generation sequencing (NGS) revealed active bacterial communities, particularly thermophilic strains in the early phase, with dominant phyla including Proteobacteria, Actinobacteria, Firmicutes, and Bacteroidetes. *Bacillus* was considered the dominant genus. The results revealed the effectiveness of co-composting in reducing PCDD/F levels, as supported by microbial activity and reactor design [139].

Biostimulation is an effective procedure for pollutant removal from soil but it has certain limitations. Providing nutrients like phosphorus (P) and nitrogen (N) leads to oxygen depletion, known as eutrophication. This enhances the growth of algae and eventually causes a reduction in the concentration of dissolved oxygen [141]. The effectiveness of the biostimulation procedure mainly depends on the different ecological factors, such as temperature, moisture content, and pH. For instance, the range of moisture contents from 2–12% by soil weight does not highly influence degradation. Furthermore, the limited availability of hydrocarbons for microbes because of the low levels of water is a disadvantage of biostimulation, and affects the biodegradation of pollutants. This limitation can be overcome through biosurfactants [142].

#### 6.1.3. Phytoremediation

Phytoremediation is a valuable technique for ecological restoration, utilizing specific plants that naturally possess the ability to clean up contaminated soil, water, and air. This technique is normally employed by wild plants characterized by unique features such as thick, rounded stems, numerous aerial roots, or extensive root systems that spread across the ground or climb the trunks of other trees. These plants can densely cover the soil surface, maintaining evergreen foliage throughout the year with minimal leaf shedding. Their high biomass production and resilience to harsh conditions in the environment make them an excellent habitat for microorganisms within the rhizosphere. This synergy creates an optimal environment for the degradation and absorption of toxic chemicals existing in the soil [143].

Phytoremediation offers a cost-effective, efficient, and eco-friendly approach for restoring an environment contaminated with pollutants, mainly for heavy metals. However, its effectiveness depends on different factors, such as the soil contamination levels, concentration of heavy metals existing in the soil, and the capacity of the plant species to effectively absorb these pollutants from the soil at the affected site [144]. Plants used in phytoremediation include hyperaccumulators, which can absorb high concentrations of pollutants but typically produce low biomass, and non-hyperaccumulators, which excrete lower amounts of metals yet create high biomass and grow more rapidly [145]. Certain plants employ different processes to eliminate heavy metals from contaminated soil, as mentioned in Figure 8C.

The processes of phytoextraction involve plants absorbing metal contaminants from the soil through their roots and transporting them to the aerial parts of the plants. Hyperaccumulator species are usually utilized for this purpose, where they absorb and store exceptionally high levels of pollutants, ranging from 100 to 1000 times more than non-hyperaccumulating plants. These plants are typically grown in the area where soil has been contaminated with different metals and POPs like dioxin for an extended period [146]. Furthermore, phytofiltration is a phytoremediation method that can take one of the three forms: rhizofiltration involves the use of plant roots, blastofiltration utilizes seedlings, and caulofiltration employs excised plant shoots for the removal of contaminants from a polluted environment [147]. Phytostimulation enhances microbiological degradation of organic pollutants through compounds that are released by plant roots. In contrast, low ethylene levels promote the growth of roots and DNA synthesis, and cell division can be inhibited by high levels. This stress is reduced by an enzyme, 1-aminocyclopropane-1-carboxylase (ACC) deaminase, which is produced by PGPR and lowers the ethylene levels. These beneficial microbes use root exudates as an energy source and also help in breaking down the contamination [148]. Phytostabilization is another process where plant roots are utilized to immobilize dioxin and other heavy metals within the rhizosphere and prevent their spreading in the environment. This process occurs through root sorption, metal reduction, valence, complexation, and precipitation. The effectiveness depends on the availability of pollutants in the soil and plant traits like deep roots and limited translocation. Plant growth can be enhanced by compost or biochar, whereas pollutants remain in the roots [149].

The processes known as phytovolatilization involve the pollutant uptake by plants and their conversion into volatile forms, which are then released into the atmosphere. An example is given in the following. Tobacco can absorb toxic pollutants like dioxin and methyl mercury from contaminated soil and convert them into less harmful forms that are released through the leaves. This process of transformation is driven by plant metabolism and supported by rhizospheric microbes [150]. Phytodegradation involves plant enzymes like dehalogenase and nitroreductase, breaking down the organic compounds into less harmful substances under optimal temperature and pH conditions. Rhizodegradation is facilitated by nutrient-rich root zones, enhancing degradation by attracting more microbes compared to bulk soil [151].

Additionally, biological products like hypothetical DECOM1, which consists of organic humus and nutrient salts, can be introduced into the soil to accelerate the breakdown of hazardous substances. This approach helps in the concentration reduction of persistent pollutants by enhancing their degradation. In other words, such products support plants in absorbing organic toxins from the soil more efficiently and effectively [152]. The phytoremediation process of persistent organic compounds in soil often requires a significant amount of time and depends on various practical factors like weather conditions, climate variations, and additional anthropogenic influences [153]. Table 6 provides a summary of the plants with the highest efficiency in removing dioxins from soil, and their effectiveness in such treatments.

The process of phytoremediation for dioxin removal from soil is scalable and cost-effective for large, polluted areas but still has some limitations. Phytoremediation is a very slow procedure for remediation purposes, as well as being limited to shallow zones, with minimal effects in deeper soil. Similarly, the procedure is unable to take up effectively the highly chlorinated dioxin and depends totally on healthy plants for effective remediation processes. This procedure is therefore best when limited to low concentrations of dioxin. The slow remediation makes the process suitable for mainly low concentrations of dioxin in shallow areas [11].

Biodegradation is a promising method for degrading dioxins in contaminated soil, as discussed above, but achieving high decomposition efficacy requires the involvement of multiple microbial strains. The success of biodegradation depends heavily on microbial activity during the process, which can be influenced by key operational parameters such as pH, incubation duration, aeration, level of moisture, and the carbon–nitrogen (C/N) ratio. These parameters must be carefully optimized based on factors such as the scale of the composting system, the properties of the soil, and the nature of the composting material. Despite its potential, this process faces challenges, including the time-intensive nature of microbial activity, which can limit its practical application at larger scales. The effectiveness of the processes also varies with the characteristics of the dioxins being treated, requiring the processes to be tailored to specific site conditions. Combining biodegradation with other treatment methods, such as the integration of chemical or physical approaches, may enhance its efficiency in dioxin degradation [9].

### 6.2. Physical Remediation Techniques

**Figure 9:**
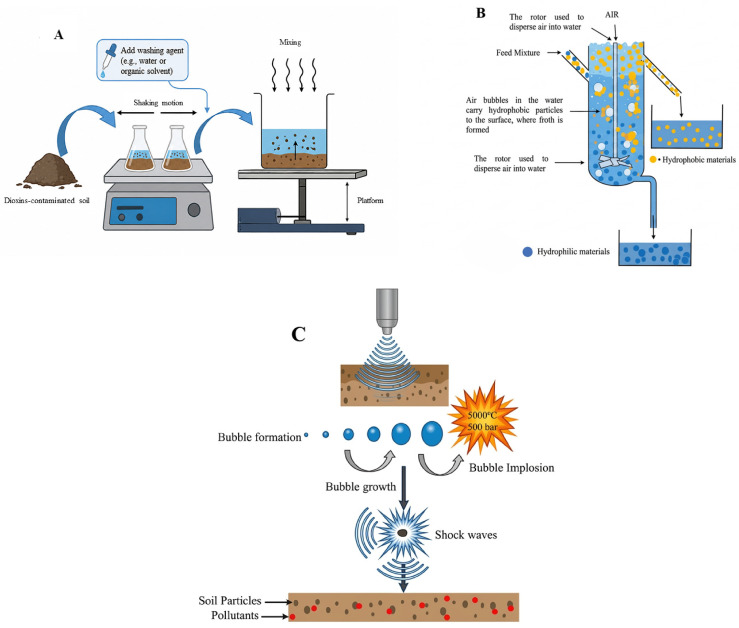
Mechanistic illustration of physical separation methods for dioxin-contaminated soil washing: (**A**) Mechanical shaking, (**B**) Froth flotation, and (**C**) Ultrasonication. BioRender was used for figure creation. BioRender: Scientific Figure and Illustration Software.

The effectiveness of washing out dioxin from soil depends on the physical method used. A visual representation of the main physical techniques, including shaking, mechanical shaking, froth floatation, and ultrasonication, along with their mechanisms, is shown in Figure 9.

The physical techniques are effective in their removal of dioxin from soil. This type of technique has many advantages, like higher mechanical strength, simple and easy operation, low energy consumption, and organic and inorganic removal at the same time, but still, the procedure has many significant limitations and faces challenges, affecting its practical applications. The procedure known as mechanical stirring is simple and cost-effective, but generates high noise because of the axial movement, requires complex installation, and requires regular maintenance. Furthermore, the procedure needs a large volume of solutions used for washing, and a large operational space is required [159].

Similarly, mechanical shaking is simple and easy to design and requires less energy but always has limitations in the upscaling of large applications and shows low competence for highly cohesive mixtures. Furthermore, low solvent usage and high efficiency for dioxin removal are offered by the ultrasonication procedure, but this procedure consumes high levels of energy, requires high levels of maintenance and has a high investment cost and an irritating noise while operating, in addition to raising the temperature. The froth flotation also needs high energy and faces challenges in controlling airflow rates and agitations. These physical procedures can create secondary waste streams, including residual solvents and contaminated wash solutions, that pose challenges regarding ecological management, and overall costs used for treatment are increased [12].

#### 6.2.1. Mechanical Shaking

Mechanical shaking is a procedure that includes orbital, reciprocating, and rotary shakers. This technique has been used to eliminate PCCD, PAHs, and PCBs from contaminated soils. This system works through linear translational motions, as mentioned in Figure 9A. The transition from laminar to turbulent flow (Reynolds number > 60,000) is desirable, as this improves the interaction between the washing agents and contaminants. Fluid viscosity and shaking speed play major roles. The mobility of the contaminant is reduced, and the removal efficiency is reduced by higher viscosity [160]. Each shaker has its unique benefits. Reciprocating shakers are commonly used due to their steady and reliable operation. Orbital shakers are often operated at low speeds (150–200 rpm) to maximize contact time [161]. Rotary shakers provide additional tilting capability (0–90°), achieving 76% removal of PCDD at 27 rpm in 10 min by using ethanol. Efficiency can also depend on setup; for example, a 24-well shaker was found to be more effective than a 96-well shaker under the same conditions. Each shaker type offers unique benefits. Reciprocating shakers are commonly used due to their steady and reliable operation [55]. Overall, mechanical shaking is favored in different environmental applications for its simplicity, low requirement of energy, and minimal solvent usage. However, the procedure is not suitable for highly cohesive soils, cannot break down aggregates, and lacks compatibility with continuous processing factors, which limits its scalability for large-scale soil washing and treatment purposes [162].

#### 6.2.2. Froth Flotation

Froth flotation is a commonly used method in mining separation and has been recently adapted to treat dioxin-contaminated soil, with good results. The techniques utilize differences in hydrophobicity to separate the organic pollutants from soil particles. Figure 9B shows the floatation mechanism, including four stages: (1) collision between contaminants and air bubbles, (2) contaminants’ attachment to bubbles, (3) rising of these bubbles and contaminants aggregate to the foam layers, and (4) subsequent detachment and removal [163]. The procedure’s effectiveness is influenced by different operational parameters such as flow rate of air, bubble size, flotation time, temperature, and agitation speed. Tran et al. [12] determined that increasing flotation time improves the removal of contaminants, though extended durations raise energy costs for agitation and air generation [12]. Higher airflow and agitation enhance contact between particles and bubbles, but excessive values can destabilize foam and increase contaminant detachment. The optimal conditions for dioxin removal were determined to be 2.5 L/min airflow and 1200 rpm agitation [164].

Small bubbles enhance the chances of particle collisions and lower the chances of detachment. The research proposes that bubbles around 1 mm in diameter are ideal for efficient flotation, as they promote better distribution of gas in turbulent conditions. Temperature also plays a key role in the interaction between pollutants and bubbles. Higher temperatures can improve the attachment of particles and bubbles, decrease the viscosity and density of hydrophobic substances, and help in the release of volatile organic compounds (VOCs). The effectiveness of the procedure of flotation is influenced by the surfactant concentration. For example, increasing the amount of cocamidopropyl betaine (BW) from 1% to 3% (*v*/*w*) significantly boosted the solubilization of PCDD, which led to better removal outcomes. In a similar case, an increase in the SDS concentration from 50 ppm to 5000 ppm resulted in an improvement in the efficiency of pollutant removal from 48% to 58% [165]. Furthermore, surfactants help in the removal of contaminants by lowering the surface tension at the soil–liquid interface, given their effectiveness, which is influenced by the pH of the washing solution. A higher pH, such as with the increased levels of NaOH, enhances dioxin solubilization by weakening its bond to soil particles. Overall, froth floatation offers effective contact between particles and cleaning agents, removes both organic and inorganic pollutants, and is accessible, though requiring high energy and precise control of operational parameters like agitation and airflow [166].

#### 6.2.3. Ultrasonication

Ultrasonication is a treatment procedure that has been used to effectively remove the PCDD/Fs from contaminated soil. This method involves two main processes: the creation and subsequent collapse of cavitation bubbles, and the release of the shock waves that result from their collapse, as shown in Figure 9C. The collapse of these bubbles generates intense local energy, resulting in two distinct effects: sonophysical and sonochemical phenomena. The sonochemical effects are mostly responsible for the degradation of contaminants through pyrolytic reactions and the formation of reactive radicals, including hydroxyl radicals. These radicals play a major role in the oxidative breakdown of organic pollutants [167]. On the other hand, sonophysical effects such as microjets, shock waves, and intense local turbulence enhance the physical processes like micromixing and the desorption of pollutants from soil particles into the aqueous phase. These effects are mainly pronounced because of their interaction with both liquid and solid phases [168].

Furthermore, the procedure has attracted significant attention for its main applications in the treatment of soil by remediating organic contaminants from soil. During this procedure, the primary mechanisms include activated oxidants, free radical generation, enhanced mass transfer, improved soil permeability, and pollutant degradation. The ultrasonic phenomena are mainly based on the hot-spot theory. Initially, in the local hot spot with a high temperature up to 5000 K, which is generated by cavitation bubbles, the breakdown of chemical bonds will occur among the adsorbent surfaces and the pollutants [169]. Secondly, shockwaves with high pressure and rapid microjets are produced by the ultrasounds in the water and soil mixture, significantly affecting the soil surfaces and increasing the desorption rate of pollutants. The reactive radicals are generated, and the high temperature microenvironment can decompose the soil pollutants nonstop, enhancing the overall mechanism of degradation. Soil characteristics such as particle size, contents of organic matter, porosity, and other properties play a key role in determining the effects of degradation procedures [170].

The application of ultrasonication generates cavitation that facilitates the release of pollutants from soil particles into the surrounding liquid (desorption), while simultaneously promoting their chemical breakdown through reactive species in the solution [171]. Furthermore, this procedure generates high localized pressure and temperature, which help in breaking down the rough soil particles into simple ones, which enhances the removal of contaminants efficiently. As compared to traditional soil washing techniques, ultrasonication operates continuously and with high stability and also uses a lower solvent volume than mechanical agitation. The system uses five times less water, achieving about 94% removal efficiency for dioxin [172]. Despite its effectiveness, the broader application of ultrasonication in large-scale operations is limited because of its high energy demand, high costs, and ongoing maintenance requirements. In addition, operational issues include high temperatures and an extreme level of noise, which present more practical challenges [173].

### 6.3. Chemical Washing

**Figure 10:**
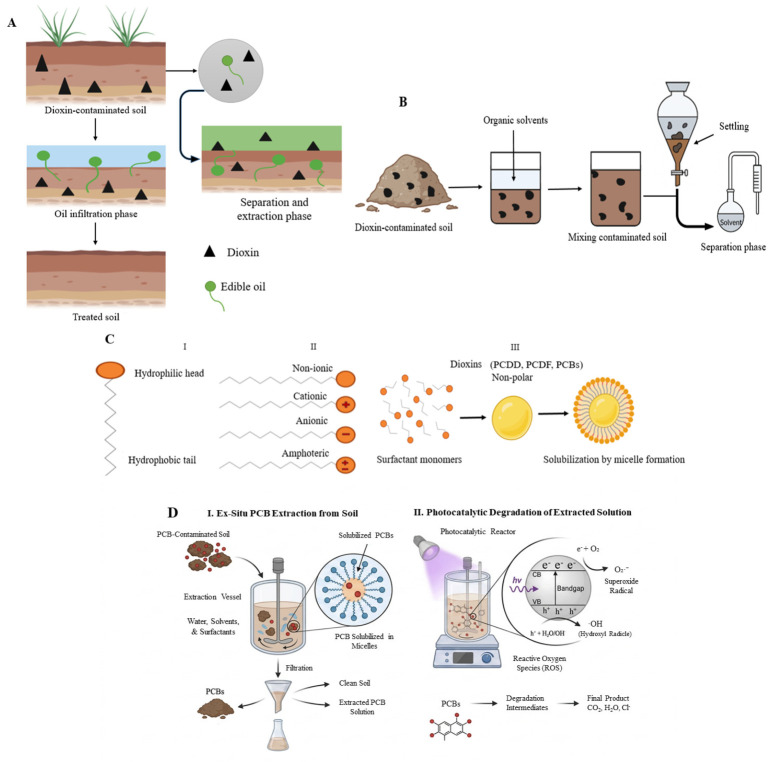

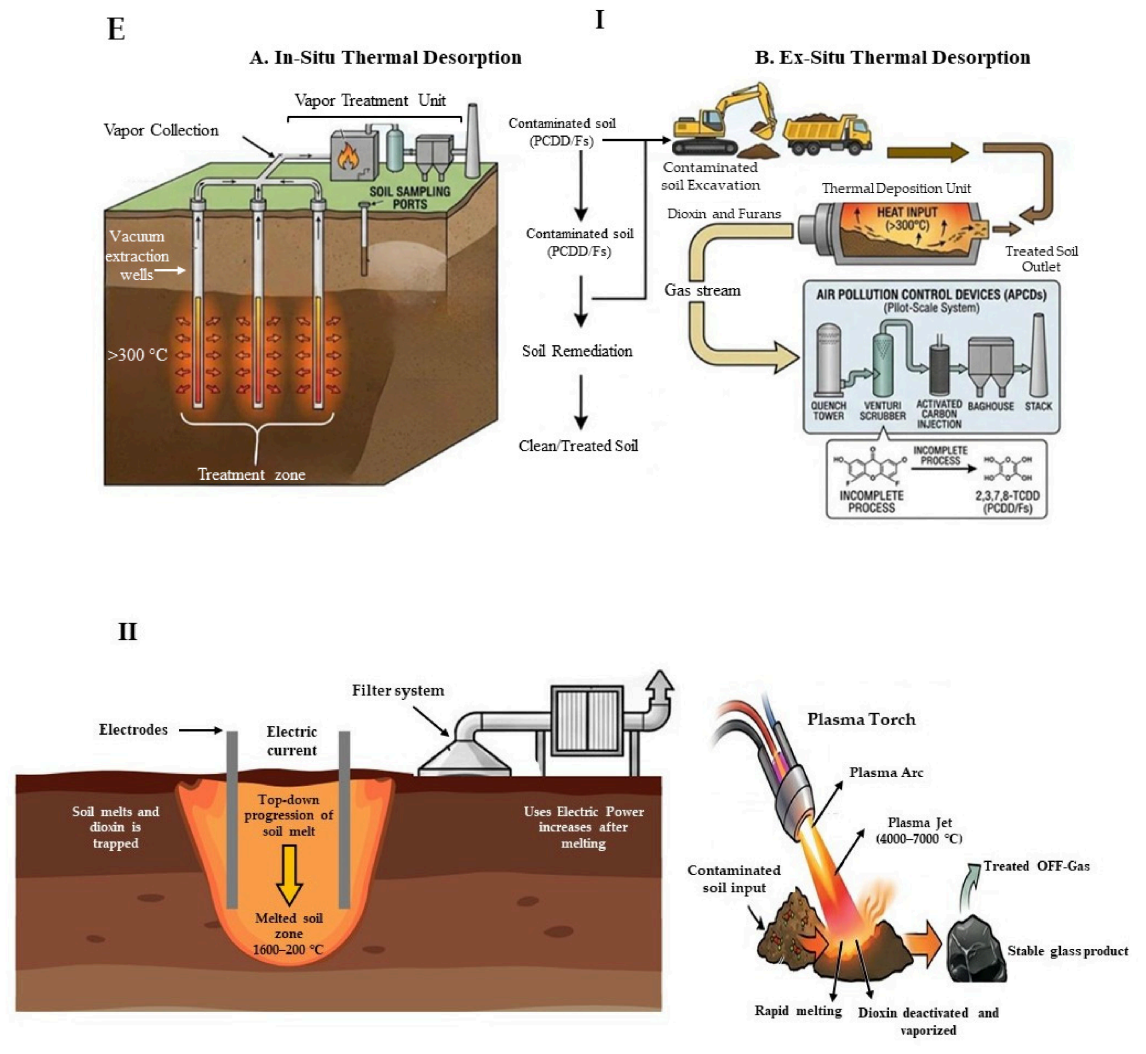
Different chemical approaches for dioxin extraction from contaminated soil. (**A**) Edible oil; (**B**) Organic solvents; (**C**) Synthetic surfactants. (**I**) Structure of synthetic surfactant, (**II**) types of synthetic surfactant, and (**III**) schematic representation of micelle formation by surfactant monomers, demonstrating their role in enhancing solubilization. (**D**) Photocatalytic remediation of PCBs; (**I**) Extraction of PCBs from soil; (**II**) Photocatalytic degradation of extracted solution. (**E**) Thermal desorption: (**I**) In situ and Ex situ thermal desorption; (**II**) Vitrification. BioRender was used for figure creation. BioRender: Scientific Figure and Illustration Software.

Chemical treatment has been widely used for the removal of dioxin from soil, as shown in Figure 10. Among them, the organic solvents are mostly used to enhance the solubilization and separation of dioxin from soil. Furthermore, surfactants have also been used to increase the desorption by reducing the surface tension and improving the contaminant mobility. Edible oils have emerged as promising alternatives, offering an eco-friendlier option for dioxin extraction from soil. These extraction procedures offer high efficiency of dioxin removal but still face many serious limitations that affect ecological and financial feasibility. The procedure, which is based on a solvent, may release toxic gases during the treatment procedure. Moreover, solvents can make negative changes in the physical and chemical properties of the soil and adversely affect the indigenous microbes in the soil. Though the edible oils are less volatile and less toxic, with high dissolution capability for PCDF/Fs, their use on a large scale is challenging because a large amount of oil is required for this procedure, which leads to high plantation demand requiring air pollution control, wastewater treatment, and industrial production [174]. The synthetic surfactants used for removal purposes exhibit high solubilization efficacy and have low toxic effects as compared to the solvents, but they are mostly expensive, less biodegradable, and can generate high organic waste as secondary pollutants, posing a high risk to soil quality [175].

#### 6.3.1. Edible Oils

Edible oils, including those from olives, vegetables, and groundnuts, can serve as a more affordable and less toxic option, compared to traditional organic solvents, for washing soil contaminated with dioxin. As organic solvents are eco-friendly, they are still considered less safe because they are man-made. There are rising concerns about their long-term impacts on human health and the ecosystem. The processes of using such oils for dioxin-contaminated soil are mentioned in Figure 10A [12]. Alternative natural solvents have also been studied, such as edible oil. Edible oils such as olive oil, vegetable oil, and groundnut oil contain triacylglyceride (TAG). TAGs are a group of aliphatic hydrocarbons with a carbon chain comprising three fatty acids; their tail can form micelles by binding with organics, which helps in the emulsification of organics. The emulsion phase of oils facilitates the solubilization of organics more than the normal phase [176].

The edible oil introduced into the soil system can act as a co-solvent, solvent, or co-solute, depending on the proportions of soil, oil, and water. When presented in higher concentration, oil acts as an immiscible organic liquid, affecting hydrophobic compounds like dioxin through cooperative mechanisms, competitive interactions, or effects related to solvents. Contaminant sorption tends to decrease due to the solvent and competitive effects, whereas it is enhanced by the cooperative effect. When the oil amount and polarity are satisfactory and sufficient, the contaminants can be released, dissolved, and removed from the soil [177]. Dioxin has a strong hydrophobic nature, due to which it partitions in the lipid stage, demonstrating solubility that is several orders of magnitude greater in oil than in water. The vegetable oil is mostly composed of triglyceride (93–99%) and acts as a light non-aqueous phase liquid (LNAPL) and exhibits partitioning characteristics similar to those of soil’s organic matter [178].

An emulsified oil solution that contained 35% edible oil, with the supplementation of surfactants and molasses, was used for removing soil contaminated with Octachlorodibenzodioxin (OCDD). The results indicated 67–74% of removal of OCDD, which can be increased by mixing with water [179]. Oil addition can dissolve the partition of soil organic matter that binds the hydrophobic contaminants, enhancing their removal or extraction. The pollutants with low molecular weight tend to partition more easily from water into soil, but the high-molecular-weight pollutants transfer primarily from soil organic matter to oil and show 66–99% dioxin-removing efficiency [12].

A study by Shelepchikov demonstrated the application of edible oils as sample matrices for solid phase extraction of dioxins and dioxin-like PCBs. The technique achieved 79 to 119% removal rates for 2,3,4,7,8-PeCDF and 2,3,7,8-TCDD. These results indicated that edible oil offers an effective recovery and quantification of dioxins with optimized solid phase extraction [180]. Additionally, oils with low viscosity tend to interact more effectively with PCDD/Fs. Studies have proved that vegetable oils can outperform solvents like hexane and toluene. Edible oils perform as natural solvents with low volatility and strong capacity for dissolving PCDD/Fs. They are cost-effective, non-toxic, and may improve the biodegradability of contaminated soil [12]. However, there are some limitations of the edible oil used for dioxin removal from soil. The use of edible oil for such treatment consumes oil on a large scale and is debated. The technique demands oil on a large scale, industrial production, and may lead to challenges in the treatment of pollutants [174].

#### 6.3.2. Organic Solvents

As PCDD/Fs are hydrophobic, non-polar compounds, organic solvents such as methanol, toluene, 2-pentanol, and ethanol can be effective in dioxin washing [90]. The general processes of soil washing with organic solvents, highlighting the main steps of mixing, settling, and phase separation, are presented in Figure 10B. Ethanol washing is a widely used solvent-based technique for dioxin washing because of its low molecular weight, low cost, and high removal efficiency. Dioxin removal efficiency from ethanol ranges from 65% to 99%. The PCDD/F removal efficiency by ethanol depends upon the organic content of soil; for example, in sandy soil, higher dioxin removal efficiency is achieved because of the low organic content of sand [181]. Different studies have shown that various types of organic solvents are used for the removal of dioxin from different soils, as shown in Table 7.

Solvents can penetrate deeper into soils with low organic content. The solubilization ability greatly depends on the type of solvent. Since dimethyl ether, ethanol, 2-propanol, and methanol are fully miscible organic solvents, they exhibit greater efficiency in solubilizing PCDD/Fs, compared to partially miscible solvents such as trichloroethylene, toluene, and xylene [184]. The cost of treatment using organic solvents can be reduced by diluting them with water. Previous studies have shown that a 75:25 (*v*/*v*) ethanol–water mixture can achieve up to 97% removal of PCDD/Fs after 10 washing cycles [181]. Although some of the solvents are considered eco-friendly, the concern remains about their effect on the ecosystem and the human body, since they are synthesized. Therefore, the use of natural solvents has also been explored alternatively. Some of the natural solvents, such as pineapple and sugarcane wine, have also been used for the removal of dioxin from soil, since they contain high amounts of alcohol [185]. In one of the studies, sugarcane wine was used for the removal of PCDD/Fs, showing great efficiency in the removal of about 60%. The processes were repeated for six cycles, and the removal of pollutants using sugar cane reached about 80%. Similarly, fish oil acts as a natural solvent because of its composition of aromatic compounds, organic acids, and alcohol, which was the dissolution of PCDD/Fs [186]. Organic solvents may be effective for the removal of dioxin from soil, but there are certain disadvantages. The process is very costly, there is high risk in the storage and handling of the solvents, and the process releases toxic gases and VOCs during the treatment and negatively affects the chemical and physical properties of soil and microorganisms [187].

#### 6.3.3. Synthetic Surfactants

Surfactants are surface-active compounds that consist of a hydrophilic head and hydrophobic tail, and they are capable of solubilizing organics. Due to their amphiphilic nature, surfactants preferentially absorb at interfaces and reduce surface and interfacial tension between immiscible phases, such as water and hydrophobic organic compounds. They minimize the surface tension of poorly soluble organic compounds by forming a micelle above a critical micelle concentration (CMC) [67]. Some studies also suggest adding salts along with surfactant for efficient washing of dioxins, as salt acts as a bridge between a hydrophobic molecule and a surfactant [188]. Synthetic surfactants can be nonionic, ionic, and amphoteric in nature, as mentioned in Figure 10C. Nonionic synthetic surfactants include Brij 35, Tween 80, and Triton X-100. While the ionic synthetic surfactants are decyl dimethyl ammonium chloride (DDAC), sodium dodecyl sulfate (SDS), sodium dodecyl benzene sulfonate (SDBS), and cetyltrimethyl ammonium bromide (CTAB), the amphoteric synthetic surfactants are cocamidopropyl hydroxysultaine (CAS) and cocamidopropyl betaine (CAPB) [189].

Nonionic solvents are considered to be better than ionic solvents because of their low cost, reduced toxicity, better solarization capacity, and low sorption ability in soil. They rarely dissociate in water, and their oxygen-based groups are present in their hydrophilic part, such as polyoxyethylene or hydroxyl. They form hydrogen bonds between water molecules and their hydrophilic phase, which are dissolved in the aqueous phase. They can easily form micelles between non-polar chains by hydrophobic interaction. For ionic synthetic surfactants, micellization is difficult, as they require surfactants in high concentration to overcome repulsion between their head groups. Non-ionic surfactants such as Triton X-100 have shown high performance in washing dioxins because of their low CMC [12]. In ionic surfactants, cationic surfactants show more affinity to adsorb on soil surfaces that are negatively charged, which reduces their volume. Anionic surfactants show high performance because of their strong bond with hydrophobic contaminants. SDS showed 81% efficiency in PCDD/F removal. Amphoteric surfactants have shown high performance because of their low CMC, high biodegradation, and stable micelle [190]. However, because of their high cost, they are less commonly used in practice and are comparatively a very complex mechanism. Their lower biodegradation leads to negative effects on the soil properties. Organic waste can be released in higher concentrations as a secondary [174,175,176], pollutant [178,179].

#### 6.3.4. Photocatalytic Remediation

Photocatalysis is the procedure where the chemical reaction is initiated or accelerated by visible, infrared, or UV light in the presence of a photocatalyst. Photocatalysts have the capability of engaging in chemical interactions with reactants, transformation facilitation, and restoration of original composition after each cycle, through continuous adsorption of infrared light, UV, and visible light [191]. In this process, the dioxins are removed through photodegradation, as shown in Figure 10D. In these advanced oxidation procedures, the ROS is generated by a light-activated catalyst such as titanium dioxide (TiO_2_), which degrades the POPs. The contaminants are normally extracted and then exposed to photocatalytic degradation in the remediation procedures of PCBs. This is required due to the hydrophobic nature of PCBs, where the direct treatment in solid wastes is limited. Different solvents and surfactants are used for the separation of PCBs from soil. Generally, ex situ methods like extraction and washing of solvents are applied by using various solvents, including acetone, isopropyl alcohol, dimethyl ether, and acetone for the suspension of contaminants [192].

The extraction efficacy mostly depends on the surfactant’s properties; it should exhibit a high capacity of solubilization and high stability, and have minimum adsorption on the soil components. Generally, the anionic surfactant has high CMC, but cationic surfactants tend to adsorb onto soil particles strongly. Upon irradiation, electron hole pairs are produced by the photocatalyst. The photo-generated holes (h^+^) react with hydroxide ions or water for the formation of hydroxyl radicals (•OH), whereas oxygen is reduced by the electron (e^−^) for the formation of superoxide radicals (O_2_•^−^). The highly reactive species initiate the PCB degradation through the means of hydroxylation, dechlorination, and mineralization [193]. As discussed earlier, solvent selection can influence the degradation of dioxin and lead to the formation of various intermediate products. For instance, the acetone used in the TiO/UV_2_/UV system led to the formation of hydroxylated chlorobiphenyl (OH-PCB) through the hydroxylation reaction; however, the use of ethanol or 2-propanol results mainly in chlorobiphenyl biphenyl formation during the photoxidation and photolysis [193]. Zhu et al. [194] treated the PCBs in contaminated soil. Two surfactants were used: polyoxymethylene lauryl ether (Brij 35) and hydroxypropyl-β-cyclodextrin (HPβCD). This was followed by photocatalytic treatment based on TiO_2_. The results obtained showed that HPβCD was found to be more effective in the extraction of PCBs from the soil, with an extraction rate of about 91.2%; Brij 35 achieved about 77.2% extraction. The photocatalytic degradation of dioxin in the extracted solution follows pseudo-first-order kinetics for both the surfactants. A high efficiency of degradation was observed in the Brij 35 solution, about 75%, as compared to the HPβCD solution, at about 39%. This shows that HPβCD improves the extraction of contaminants and may inhibit the processes of photocatalytic degradation, affecting the efficiency of overall breakdown processes [194].

These techniques have several limitations, such as the need for extraction of pollutants before the degradation, making this unsuitable for in situ remediation. The procedure also leads to the production of secondary wastes that need further treatment. Furthermore, the effectiveness is very sensitive to the different kinds of surfactants and solvents used. Incomplete degradation can result in the generation of intermediate products, causing serious ecological risks [195].

#### 6.3.5. Thermal Desorption

Thermal desorption is recognized as an effective ex situ remediation procedure for treating dioxin-contaminated soil. The techniques utilize heat to promote the vaporization of dioxin in the soil. The soil surface is heated above 300 °C by using a heat element to evaporate dioxins and furans from the soil [196]. Mostly, thermal remediation procedures are carried out in two ways, such as in situ and ex situ thermal desorption, as mentioned in Figure 10E (I). This procedure is considered one of the fastest and most reliable techniques for the remediation of soil, but the high temperature can affect the soil’s properties, mostly its density and compaction. Thermal remediation based on the zone combustion procedure has been proposed for the treatment of dioxin-contaminated soil [197]. According to Troxler et al. [198], the thermal desorption method has been used for the treatment of ∼175,000 tonnes of soil contaminated with dioxin [198]. Rathna et al. [197] developed a pilot-scale thermal system. This system was equipped with air pollution control devices for the treatment of PCDD/F-contaminated soil. The 2,3,7,8-TCDD was produced due to the incomplete process of dichlorination. So, further studies are needed on degradation of each chemical [197].

Vitrification is also a thermal procedure used for the treatment of dioxin-contaminated soil. The techniques are carried out at a very high temperature ranging from 1600–2000 °C. In this procedure, the soil is melted at a very high temperature. The soil is then cooled to form a hard, chemically stable glass with low potential of leaching. The pollutants are encapsulated into the glass matrix, preventing the leaching of toxic compounds into the environment, as mentioned in Figure 10E(II) [199]. In this procedure, electrical power is used to provide heat for the treatment of soil. The top layer of soil melts and becomes an electrical conductor after passing the electric current. In this way, electricity and heat are provided to the deep layer of the soil. The soil melts and the contaminants are trapped. The procedures are very costly and consume high energy. In the processes of plasma vitrification, a plasma torch is used to melt the dioxin-contaminated soil. Temperatures in the range of 4000–7000 °C can be achieved rapidly through this procedure. The dioxins and furans become completely deactivated and vaporized due to high temperature. Therefore, the concentration of dioxin is reduced in the soil [197].

Even given its effectiveness, the procedure has several limitations. The treatment efficacy is influenced by site characteristics and the type of soil, like its moisture and texture, and the level of contamination. The processes are very expensive, and high levels of energy are required to obtain the temperature required for soil melting. Additionally, vitrification can change the natural properties of soil, including structure, fertility, and compaction, and lead to the limited reuse after the treatment [197].

The shortcomings of the individual capabilities of physical, chemical, and biological remediation reveal the need to take a blended, step-by-step approach. They cannot be applied universally, but their combination with a strategic approach can address their weaknesses separately and further improve the overall performance as well as make the results sustainable. The next section suggests a systematic model of application for such an integrated remediation strategy implementation.

## 7. An Integrated Strategy for Sustainable Remediation of Dioxin

**Figure 11:**
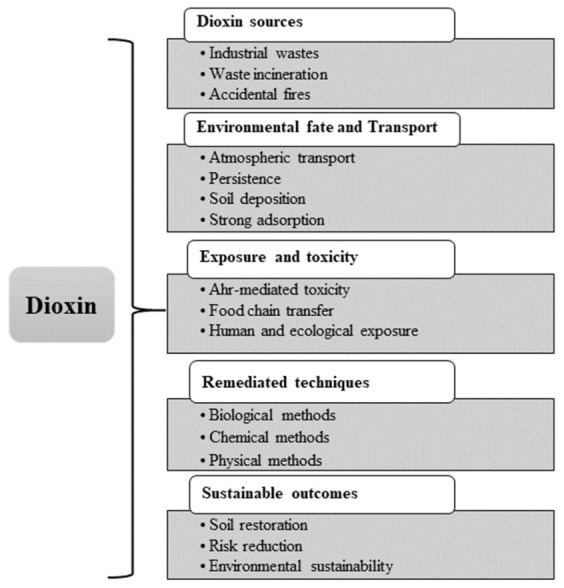
Showing the framework illustrating the sources, environmental contamination, toxicity, and remediation techniques associated with dioxin.

A sustainable and effective framework of dioxin remediation requires consideration of the complete life-cycle of these pollutants, such as their sources of emission, places of environmental contamination, toxic effects, and associated health risks, instead of depending on single treatment techniques. This is due to dioxin being a highly persistent pollutant, its strong hydrophobic nature, and its tendency to accumulate in living organisms. Dioxin cannot be managed through isolated interference. The planned integration of chemical, biological, and physical techniques of remediation is required to ensure ecological and human health protection for a long time. The integration shows the different dioxin sources, exposure pathways, remediation procedures, environmental contamination and transport, and sustainable results, as mentioned in Figure 11.

Dioxins are mainly produced through different anthropogenic activities, including high-temperature industrial combustion procedures, improper disposal of hazardous wastes, waste incineration, chemical production with chlorine, and metallurgical processes. The contaminated soil, sediments, landfill sites, and fly ash deposits act as secondary sources of contamination and continuously release dioxin into the surrounding environment through volatile resuspension of particles and leaching procedures. The other natural sources, such as volcanic activity and forest fires, make minor contributions to dioxin pollution. Dioxin strongly binds to soil after being released into the environment, shows low solubility in water, is resistant to degradation, and persists in the soil for a long time. The long-distance atmospheric transport makes the dioxin remediation more challenging, since the dioxin can settle in the soil far away from where it was first released. Such characteristics need different strategies of remediation that are mainly designed according to the contaminant’s behaviors and specific conditions.

The toxic effect of dioxin is mainly exerted by binding activation and its binding to AhR, which can trigger the disruption of endocrine systems, oxidative stress, immunotoxicity, and an increased risk of cancer. The toxic effect of dioxin can occur even in small amounts. The cleanup procedure should focus on reducing the risk to a safe level instead of trying to eliminate the contaminants completely. The approach for toxic equivalency (TEQ) is essential for evaluating the site risk and prioritizing remediation, but it should be combined with the biological and ecological studies to show the actual pathways in a better way.

The biological, chemical, and physical techniques vary in their sustainability, mechanisms, and effectiveness in dioxin remediation. Physical techniques of remediation, such as washing of soil, excavation, ultrasonication, and thermal remediation, can lower the dioxin level and are mainly suitable for regions contaminated with dioxin at a high level. However, the procedure consumes energy at a very high level, disturbs the soil, and produces extra waste that is difficult to manage and is considered very expensive. Therefore, the physical remediation procedure is better for temporary and targeted measures as compared to sustainable remediation for the long term.

Chemical remediation techniques, such as chemical reduction or oxidation and surfactant-assisted washing extraction, enhance the desorption and elimination of dioxin from the soil particles. Although these techniques improve the removal efficiency, they are linked to high consumption of chemicals, which may lead to extra pollution, and do not remove the contaminant completely. Therefore, chemical procedures are more effective when used as pretreatment strategies to reduce the level of contamination and make dioxin more accessible for subsequent treatment.

Alternatively, the biological techniques used for remediation, such as phytoremediation, biostimulation, and bioaugmentation, are known to be sustainable and are the most effective remediation approaches for the treatment of dioxin-contaminated soil. The techniques that detoxify and degrade dioxin using microbial enzymatic transformation and dechlorination lead to permanent reductions in risks. These procedures work in moderate ecological conditions, maintain the structure of soil and ecological functions, have low energy levels, and minimize the production of extra wastes. Although the biological procedure normally takes a long time and proceeds slowly as compared to chemical and physical techniques, this procedure provides environmental restoration and detoxification for a long time, which makes this procedure the most suitable and sustainable one for remediation of dioxin-contaminated soil. Study has determined that the most effective methods for the remediation of soil contaminated with dioxin involve stepwise and combined strategies in which the chemical and physical techniques are used initially to lower the level of contaminants and improve the convenience and accessibility for subsequent biological treatments using the primary techniques for long-duration treatment of dioxin-contaminated soil.

### 7.1. A Stepwise Integrated Framework for Sustainable Dioxin Remediation

The proper and viable remediation of dioxin-contaminated soil should be based on a multiphase, integrated strategy based on ecological safety, long-term effectiveness, and economic viability. Considering the evaluated methods and their drawbacks, we suggest a step-by-step structure in which physical, chemical, and biological approaches are used in a rational order and, hence, are allowed to obtain the maximum contaminant removal and elimination. Performing this step requires site procurement, a risk assessment of the location, and a description of that location, according to established planning and project management standards.

#### 7.1.1. Step 1: Site Characterization and Risk Assessment

Prior to any intervention, site assessment should provide information on the level of dioxin in soils (e.g., the content of organic matters, texture, pH, bioavailability, and possible routes of exposure). This step will assist in informing the selection of relevant technologies and setting up cleanup objectives relevant to risk-based target levels (RBTLs) and other applicable guidelines (e.g., WHO, EPA).

#### 7.1.2. Step 2: Primary Treatment Physical and Chemical Mobilization

In soils containing high levels of dioxin (>1000 ppt of TEQ), physical or chemical pretreatment is recommended to dilute the contaminant load so that successful treatment can be achieved by biological methods. The options include (1) washing the soil with environmentally friendly solvents (e.g., ethanol–water mixtures) or even with edible oils; (2) extraction of dioxins in soil particles using surfactant; and (3) granular soils with low fines can be separated physically (e.g., froth flotation).

These approaches decrease the total mass of contaminants and cause heretofore robust soil–dioxin bonds to be ready to undergo biological treatment.

#### 7.1.3. Step 3 Secondary Treatment: Biological Detoxification

After the initial decontamination, biological means are used to remove the rest of the dioxins and decrease the danger in the long term: (1) microbial consortia bioaugmentation (e.g., *Pseudomonas* spp., *Dehalococcoides*) targeted to the local conditions; (2) biostimulation by nutrient amendment (e.g., C/N optimization) to stimulate native microbial activity; and (3) residual contamination in shallow soils for phytoremediation with hyperaccumulator plants (e.g., zucchini, rice). Incorporating biological treatments encourages individual detoxification by accomplishing dechlorination using microorganisms and breaking substances down with the help of enzymes, approaches which are in line with the sustainability objectives.

#### 7.1.4. Step 4: Monitoring and Verification of Treatment

Long-term monitoring should be done to confirm the decrease in the dioxin levels and ecological recovery after remediation. The parameters include (1) bioavailability and level of soil TEQ, (2) composition and diversity of microbial community, and (3) in the areas treated, plant health and ecosystem functioning.

Follow-ups will be used to verify the efficiency of the treatment and will give information on adaptive management in case of necessity.

#### 7.1.5. Step 5: Reclamation and Reuse of Land and Restoration Biology

The last stage is the restoration of soil structure and functionality by the use of organic amendments (e.g., compost, biochar) and re-vegetation using native vegetation. This will improve the fertility of the soil, stabilize the existing contaminants, and make the land safe with respect to reuses involving farm, recreational, or ecological purposes.

**Figure 12:**
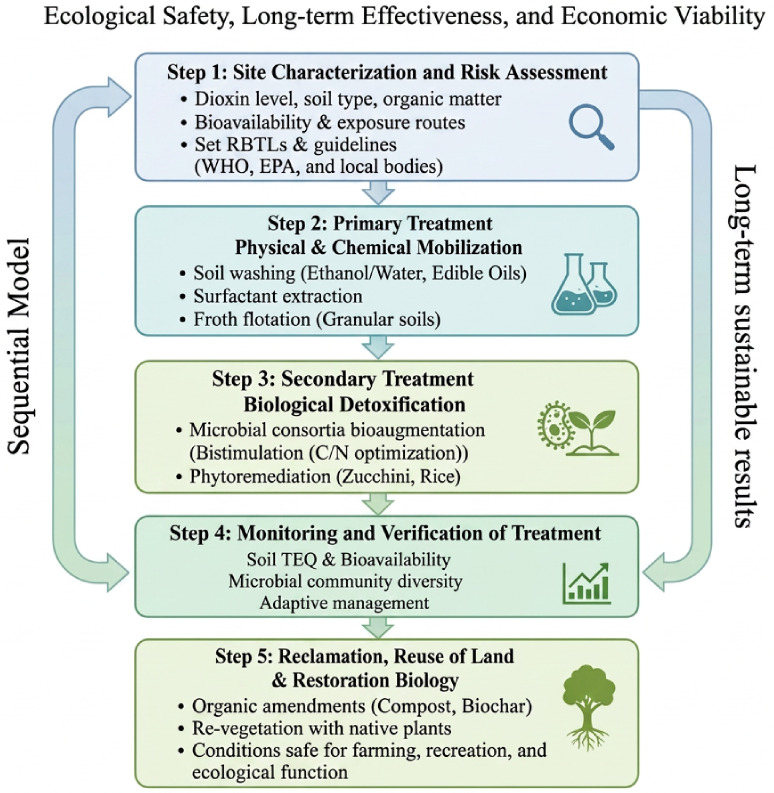
A stepwise integrated framework for sustainable dioxin remediation. BioRender was used for figure creation. BioRender: Scientific Figure and Illustration Software.

This sequential model presents a logical framework (Figure 12) for the remediation of dioxin soils, featuring the equitable incorporation of technologies to deliver sustainable, long-term, and green results.

## 8. Conclusions and Future Perspectives

The step-by-step integrated framework proposed places emphasis on the shift from disjointed efforts in remediation to the holistic systems approach. Such a strategy combines the advantages of the physical, chemical, and biological techniques, not only to deal with the instant contamination, but also to encourage the sustainability of the soil and ecosystem in the long run. Such multi-level, adaptive frameworks should be used in future remediation efforts to increase flexibility, economic viability, and environmental accountability. Dioxins are extremely persistent and toxic pollutants that present a serious threat to human health and the environment. Their low water solubility, resistance to degradation, and chemical stability allow them to accumulate in soil and magnify through food chains, resulting in contamination and adverse health effects, including cancer, endocrine system disruption, and neurological damage. Industrial activities and combustion processes contribute to more than 80% of global dioxin emissions, with soil functioning as a key repository for these contaminants.

This review highlights the different sources and pathways through which dioxins are produced, travel, accumulate, and persist in the environment. As discussed earlier, sustainable remediation of dioxin from the soil does not only depend on the chemical, physical, and biological procedures, but also requires an integrated framework of remediation within which long-term stability, toxicity, and pollutant behavior is addressed. Amongst the evaluated methods, the promising biological remediation techniques, such as bioaugmentation, biostimulation, and phytoremediation, have demonstrated success in reducing levels of dioxin. For example, microorganisms like *Pseudomonas* spp. and fungi such as *Phanerochaete chrysosporium* can degrade dioxins effectively, while plants like zucchini and rice have shown significant potential for the absorption of these pollutants. Furthermore, physical strategies, including mechanical shaking, froth flotation, and ultrasonication, can improve contaminant separation efficiency. In addition, chemical treatment employing edible oils, synthetic surfactants, organic solvents, photocatalytic remediation, and thermal desorption have demonstrated potential in mobilizing and degrading dioxin in soil and supporting subsequent biological degradation. Surfactants can effectively promote the removal efficiency of dioxin, but must be used carefully due to the risks of secondary pollutants. Challenges remain with these strategies as to field use and optimizing them under diverse ecological conditions.

All the remediation techniques used for the treatment of soil contaminated with dioxin have limitations and drawbacks. Specific attention should be given to the secondary pollutants caused by the VOCs, like ethanol and methanol, and the release of organic waste during the procedure of remediation. The release of VOCs is a serious matter and should be a focus for future research activities. These pollutant compounds affect microbial communities and the structure of soil. The biological remediation techniques provide more sustainable and eco-friendly solutions. This technique degrades and detoxifies the dioxin directly through enzymatic transformation and different microbial activities, leading to long-term risk reduction rather than simple transfer of pollutants. Although biological treatment is a slow procedure and may take a longer time for degradation, it consumes less energy, minimizes secondary pollutants, and preserves the structure of the soil. These treatment procedures have emerged as the eco-friendly and most sustainable approach. Effective dioxin management requires a comprehensive approach that combines advanced remediation technologies, continuous monitoring, and robust regulatory policies. Integration of physicochemical and bio-based approaches, including surfactant washing with phytoremediation and bioaugmentation, can support the long-term restoration of soil. There is an urgent need for global collaboration to address dioxin contamination, given its persistence and severe health implications, as well as the development of an advanced detection technique that is both cost-effective and time-efficient. Advanced technologies can be considered in future studies, including nanomaterials, electrokinetics, and nano–enzyme remediation systems. The impact of climate change on the contamination and ecological behavior of dioxin is another critical perspective. The temperature rise, changes in the pattern of rainfall, redox conditions, and changes in soil moisture can highly affect the rate of dioxin degradation, volatilization, and mobility. Therefore, designing climate-resistant remediation strategies and applying predictive modeling for various climate conditions is important in maintaining long-term stability and minimizing the risk of exposure.

Future research should focus on the bioavailable fraction of dioxin rather than its total concentration. This fraction controls the ecological risks and the successes of remediation procedures. Advanced biological and analytical methods need to be developed to precisely evaluate and target these bioavailable pools. Eventually, combining biological and chemical remediation techniques, such as combining surfactants or electrokinetic procedures with plant-based procedures or microbes, is vital for achieving field-relevant, sustainable, and more effective results. Future efforts should focus on cost-effective and enhanced biological, physical, and chemical remediation methods, as well as on reducing the economic challenges in large-scale applications. A combined interdisciplinary focus on climate studies, environmental modeling, and material sciences is important for the development of sustainable and adaptive strategies for dioxin globally. The research studies in the future should focus on microbial behavior, the use of enzymes, and nanomaterials in order to enhance the rapid degradation of dioxin-like pollutants in the soil. These steps are critical to minimizing the health and environmental impacts of this persistent pollutant.

## Figures and Tables

**Table 1:** Different studies on dioxins as a pollutant.

S. No	Title	Scope	Ref.
1	Dioxin sources and current remediation technologies	Reported major sources of dioxins and remediation technologies for dioxins.	[7]
2	Ecological threats of dioxin in soil	Described environmental behavior, persistence, exposure pathways, and major health and ecological risks.	[6]
3	Human exposure to dioxins in soil	Investigated the relationship of residence and dioxin-contaminated soil.	[8]
4	A Review of Soil Contaminated with Dioxins and Biodegradation Technologies: Current Status and Future Prospects	Explains the sources, risks, and occurrences of dioxin pollution in soil globally; remediation techniques are also discussed.	[9]
5	Dioxin impacts on lipid metabolism of soil microbes: towards effective detection and bioassessment strategies	Reported the impact of dioxins on the lipid metabolism of soil microbes and their application in detection and risk assessment.	[10]
6	A review of advanced bioremediation technologies for dioxin-contaminated soil treatment: Current and Future outlook	Summarized bioremediation approaches, which include biosurfactants, phytoremediation, and composting.	[11]
7	Soil washing for the remediation of dioxin-contaminated soil: A review	Evaluated the washing of soil as a technique for the remediation of soil contaminated with dioxins.	[12]
8	A review of background dioxin concentrations in urban/suburban and rural soils across the United States: implications for site assessments and the establishment of soil cleanup levels	Reviewed the levels of dioxin compounds in rural, urban, and suburban soil in the U.S. and compared them with safety goals for future regulations.	[13]
9	Facilitated transport of dioxins in soil following unintentional release of pesticide-surfactant formulations	Investigated surfactant-enhanced transport and mobility in soil after the spill of pesticides.	[14]
10	Dioxins as priority environmental pollutants in soil: sources, risk assessment, and mitigation strategies	This study provided a detailed overview of management strategies and associated health effects. The review highlighted the linkages between soil contamination, food chain transfer, and AhR-mediated toxicity, emphasizing the potential risks to environmental and human health. Furthermore, biological, chemical, and physical techniques of remediation were reviewed.	Current study

**Table 2:** Average dioxin concentration as measured in soil samples in ppt-TEQ (parts per trillion as toxic equivalent), across different regions.

Region	Soil Type	Sources	Concentration (ppt-TEQ)	Ref.
1	Soil	Highly mountainous area	2.48–4.30	[44]
2	Soil	Urban area	8.2	[45]
3	Topsoil	Industrial site	0.34–18.05	[46]
4	Surface soil	Incineration plant	>1000	[47]
5	Soil	Urban area	475.48–3039.27	[48]
6	Soil	Industrial area	77.73	[49]
7	Soil	Agricultural area	0.05–23	[50]
8	Paddy soil	Agricultural area	18,000–540,000	[51]
9	Soil	Contaminated sawmill area	0.62–690,000	[52]
10	Topsoil	Coastal areas	14.2–27	[53]
11	Soil	Alluvial flood plain of a river	7680	[54]
12	Soil	Pentachlorophenol manufacturing factory	606,000	[55]
13	Topsoil	Highly industrial zones	0.33–9.99	[56]

**Table 3:** Toxic effects of dioxin-related compounds.

Health Effects	Dioxin Compound	Ref.
Fatigue and discomfort	PCDFs/PCBs	[81]
Meibomian gland hypersecretion	PCBs	[82]
Hypomineralization of enamel in the permanent 1st molar of children	2,3,7,8-TCDD	[83]
Pruritis	PCDFs/PCBs	[81]
Thyroid	2,3,7,8-TCDD	[84]
Immune deficiency	PCB congeners (118, 138, 153, 180) PCDD/F	[85]
Hyperpigmented conjunctivae	PCBs	[82]
Headache	PCDFs/PCBs	[81]
Peripheral and central nervous system pathology	PCDFs/PCBs	[81]
Defects in development		
Defects in reproduction		

**Table 4:** Different microbial strains with the capability to biodegrade dioxins in soil.

Microorganisms	Dioxin Compounds	Concentration	Removal (%)	Time (Days)	Ref.
*Pseudomonas* ssp. Strain CA10	2-CDD	1 µg/mL	97	5	[123]
*Rhodococcusopacus* SAO101	Dioxin(DD) 1-CDD	1 ppm	97 92	7	[124]
*Hydrocarboniphaga* BHBi4	2,3,7,8-TCDD	170 ng/g	59.1	21	[125]
*Phlebia radiata* strain 267	2,3,7,8-T4CDF 1,2,3,4,7,8-H6CDD	-	60 28	30	[126]
*Pseudallescheria boydii*	2,3,7,8-TCDD	125 ng/g	92	15	[127]
*Pseudomonas aeruginosa*	DBF 3,6-DCDF	10 mg/L	90 60	5	[128]
*Cordyceps sinensis* strain A	2,3,7-CDD DD	-	50 50	4	[129]
*Acremonium* sp. Strain 622	T4CDD H7CDD O8CDD		73 76 88	1	[9]

**Table 5:** Overview of dioxin degradation statistics in contaminated soil.

Materials	Mechanical Components	Initial Concentration	Conditions	Removal (%)	Time (Days)	Ref.
Wood chips, Bark chips, and Straw manure	Sandy loam	300–660 ng-TEQ/kg	Semi-aerobic	75	175	[121]
Compost and Wood chips	Sandy	840–5300 ng-TEQ/kg	Semi-aerobic	85	360	[138]
Animals’ manure, Sewage sludge, and Leaves	Sandy loam	88.8–912.7 mol/kg	Aerobic	32.5 61.2 36.8	42	[23]
Sawdust, Compost, and Food waste	Sandy loam	16,004 ng-TEQ/kg	Aerobic	75	42	[139]
Compost, Food waste, and sawdust	Sandy loam	6048 ng-TEQ/kg	Aerobic	70	49	[140]

**Table 6:** Dioxin degradation by phytoremediation.

Plant	Dioxin Compounds	Concentration	Removal (%)	Time (Days)	Ref.
Garland Chrysanthemum	Total PCDDs	0.543 ppt	36.1	-	[154]
*Arabidopsis thaliana*	TCDD	100 10 50	55 72 58	30	[65]
Spinach	Total PCDDs	3.42 ppt	48.6	-	[154]
Gold Rush	Total PCDDs	45 ppt-TEQ	60	32	[155]
Pumpkin Zucchini	2,3,7,8-TeCDD	0.0064 TSCF 0.0089 TSCF	79 70	4	[156]
BlackBeauty	Total PCDDs	43 ppt-TEQ	46	32	[155]
Rice paddy chaff Rice stem and leaf	Total dioxin	44 ppt 317 ppt	98 90	-	[157]
Atena Polka	PCDD	7 ppt-TEQdw	66	35	[158]
Mitsuba	Total PCDDs	0.765 ppt	38	-	[154]

**Table 7:** Organic solvents used for dioxin removal from soil.

Organic Solvent	Soil Type	Removal Efficiency	Ref.
Ethanol	Highly contaminated Sandy-silty	97%	[181]
Ethanol	Lightly contaminated Sandy-silty	81%	
Ethanol	Highly contaminated Clay soil	85%	
Methanol (Ultrasound and mechanical mixing)	Fresh soil	90%	[182]
Methanol (Ultrasound and mechanical mixing)	Weathered soil	70%	
Ethanol–Water mixture (80:20)	Contaminated soil from an incinerator	100%	[183]

## Data Availability

No new data were created or analyzed in this study.

## Abbreviations

The following abbreviations are used in this manuscript:

AhR	Aryl hydrocarbon receptor
PCDDs	Polychlorinated dibenzo-p-dioxins
PCBs	Polychlorinated biphenyls
PCDFs	Polychlorinated dibenzofurans
TCD	Tetrachlorodibenzo-p-dioxin
PVC	Polyvinyl chloride
FBC	Fluidized bed combustion boilers
I-TEF	International toxicity equivalence factor
UNEP	United Nations Environmental Programme
LRAT	Long-range atmospheric transport
ppt-TEQ	Parts per trillion as toxic equivalent
DLC	Dioxin-like chemicals
WHO	World Health Organization
EPA	Environmental Protection Agency
XRE	Xenobiotic responsive elements
AhRR	AhR repressor
ERE	Estrogen-responsive elements
REP	Relative effect potency
ToMO	Toluene-o-xylene monooxygenase
GMMs	Genetically modified microbes
POPs	Persistent organic pollutants
MNA	Monitored natural attenuation
NGS	Next-generation sequencing
ACC	Aminocyclopropane-1-carboxylase
TAG	Triacylglyceride
OCDD	Octachlorodibenzodioxin
VOCs	Volatile organic compounds
DDAC	Decyl dimethyl ammonium chloride
SDS	Sodium dodecyl sulfate
SDBS	Sodium dodecyl benzene sulfonate
CTAB	Cetyltrimethyl ammonium bromide
CAS	Cocamidopropyl hydroxysultaine
CAPB	Cocamidopropyl betaine
